# Proteinase-activated receptors (PARs) – focus on receptor-receptor-interactions and their physiological and pathophysiological impact

**DOI:** 10.1186/1478-811X-11-86

**Published:** 2013-11-11

**Authors:** Frank Gieseler, Hendrik Ungefroren, Utz Settmacher, Morley D Hollenberg, Roland Kaufmann

**Affiliations:** 1 First Department of Medicine, University Hospital Schleswig-Holstein (UKSH) Campus Lübeck, D-23538, Lübeck, Germany; 2 Department of General, Visceral and Vascular Surgery, Experimental Transplantation Surgery, Jena University Hospital, Drackendorfer Str. 1, D-07747, Jena, Germany; 3 Department of Physiology & Pharmacology, and Department of Medicine, University of Calgary, Faculty of Medicine, 3330 Hospital Drive N.W.,Calgary, Alberta T2N 4N1, Canada

## Abstract

Proteinase-activated receptors (PARs) are a subfamily of G protein-coupled receptors (GPCRs) with four members, PAR_1_, PAR_2_, PAR_3_ and PAR_4_, playing critical functions in hemostasis, thrombosis, embryonic development, wound healing, inflammation and cancer progression. PARs are characterized by a unique activation mechanism involving receptor cleavage by different proteinases at specific sites within the extracellular amino-terminus and the exposure of amino-terminal “tethered ligand“ domains that bind to and activate the cleaved receptors. After activation, the PAR family members are able to stimulate complex intracellular signalling networks via classical G protein-mediated pathways and beta-arrestin signalling. In addition, different receptor crosstalk mechanisms critically contribute to a high diversity of PAR signal transduction and receptor-trafficking processes that result in multiple physiological effects.

In this review, we summarize current information about PAR-initiated physical and functional receptor interactions and their physiological and pathological roles. We focus especially on PAR homo- and heterodimerization, transactivation of receptor tyrosine kinases (RTKs) and receptor serine/threonine kinases (RSTKs), communication with other GPCRs, toll-like receptors and NOD-like receptors, ion channel receptors, and on PAR association with cargo receptors. In addition, we discuss the suitability of these receptor interaction mechanisms as targets for modulating PAR signalling in disease.

## Proteinase-activated receptors (PARs)^1^ - a unique family of G-protein coupled receptors

PARs comprise a class A G protein-coupled receptor (GPCR) family with currently four members, PAR_1_, PAR_2_, PAR_3_ and PAR_4_[1,2] that mediate the cellular effects of proteinases (for reviews see: [3-7]). PAR_1_, PAR_3_ and PAR_4_ are main targets for the coagulation enzyme thrombin, but numerous other proteinases have been shown to cleave and activate PAR_1_ including factor Xa, plasmin, kallikreins, activated protein C (APC), matrix metalloproteinase-1 (MMP1), neutrophil elastase (NE), and neutrophil proteinase-3 (PR3). As will be seen, this activation can result from exposure of a variety of ‘tethered ligands’ that, as summarized below, can drive a variety of signalling pathways. PAR_2_, like PAR_1_, can also be activated by many serine proteinases including trypsin, neutrophil elastase, neutrophil proteinase 3, mast cell tryptase, tissue factor/factor VIIa/factor Xa, human kallikrein-related peptidases (KLKs) and membrane-tethered serine proteinase-1/matriptase 1 as well as by parasite cysteine proteinase, but is insensitive to thrombin [6].

### PARs exhibit an unusual activation mechanism

**Figure 1:**
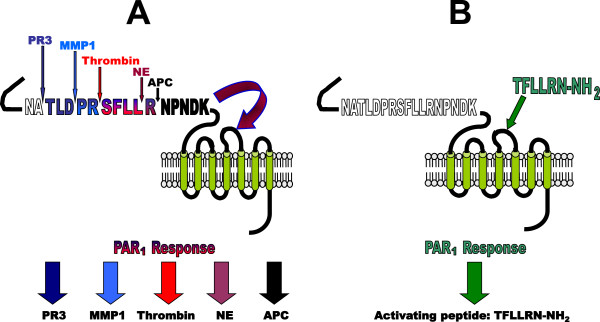
**Model for activation of PAR**_**1.**_ The scheme illustrates activation of the intact receptor by distinct mechanisms involving either proteolysis (left) or a synthetic PAR_1_-activating peptide (right): **(A)** proteolysis unmasks a tethered receptor-activating ligand (TL) sequence. The classical ‘canonical’ PAR_1_ TL sequence generated by thrombin is: SFLLRN--- [10]. Distinct ‘non-canonical’ receptor-activating TL sequences are also generated by neutrophil proteinase-3 (PR3: TLDPR---) [11], matrix metalloproteinase-1 (MMP1: PRSFLL---) [12,13], neutrophil elastase (NE: RNPNDK---) [11], and activated protein-C (APC: NPNDK---) [14,15]. The different proteinase-revealed TLs can drive very distinct signal pathways (distinct coloured arrows for PAR_1_ response at the bottom). **(B)** synthetic peptides with sequences that mimic the tethered ligand (e.g. TFLLRN-NH_2_ for PAR_1_) can activate PAR signalling without the need for receptor proteolysis. Peptides derived from the different enzyme-revealed tethered ligand sequences can stimulate ‘biased signaling’. (Illustration modified with permission from Hollenberg & Compton, Ref. [2]).

Although the PAR family members share basic structural features of all GPCRs, including a central core domain composed of seven transmembrane helices (TM-I through TM-VII) connected by three intracellular (il1, il2, and il3) and three extracellular loops (el1, el2, and el3) [8], they exhibit a unique mechanism of proteolytic activation. While most GPCRs are activated reversibly by small hydrophilic molecules to elicit cellular responses [9], PAR activation by endogenous proteinases involves the unmasking of an N-terminal ‘tethered ligand’ (TL) that remains attached to the receptor and cannot diffuse away [1-7]. Serine proteinases, such as thrombin or trypsin, are able to cleave PARs 1, 2 and 4 at specific recognition sites in the extracellular N-terminus (see Figure 1 for PAR_1_ activation). The unmasked amino terminus, functioning as a tethered ligand (curved arrow, Figure 1A), then binds to the extracellular receptor domains to trigger conformational changes and signalling.

Comparable cleavage of the N-terminus of PAR_3_ also exposes a potential “tethered ligand”, but the ability of the cleaved receptor to signal on its own is unclear. Instead, it appears that PAR_3_ acts as a cofactor for PAR_4_ activation by thrombin [16], although ‘autonomous’ signalling by PAR_3_ has been reported in a select circumstance [17]. As an alternative, PARs can be activated via proteinases by a ‘non-canonical’ mechanism involving cleavage at a site distinct from the arginine target that reveals a ‘canonical’ “tethered ligand” motif (Figure 1A). For example, MMP1 [12,13] and activated protein C (APC; [18]) can cleave the N-terminal domain of PAR_1_ to unmask a ‘non-canonical’ tethered activating sequence different from the one revealed by serine proteinases (SFLLRNPNDK…, Figure 1A). As illustrated explicitly in Figure 1A, PAR_1_ can also be cleaved by the neutrophil enzymes, proteinase-3 (PR3) and elastase (NE) to reveal receptor-activating sequences that differ not only from each other but also from those resulting from the action of MMP1 and APC [11]. Of importance these ‘non-canonical’ tethered ligands dock with the receptor to drive distinct biased signalling pathways (e.g. via MAPK but not calcium). As a further unexpected example, neutrophil elastase (NE) has recently been shown to activate PAR_2_ signalling in a ‘biased’ manner, by exposing yet another ‘non-canonical’ PAR_2_ tethered ligand sequence that selectively stimulates a mitogen-activated protein kinase (MAPK) pathway, without triggering an elevation in intracellular calcium levels as is caused by a ‘canonical’ trypsin-exposed PAR_2_ tethered ligand [14]. Finally, when the first ‘thrombin receptor’ was cloned (now termed, PAR_1_/F2R: [10,19]), it was established, that, in addition to proteinase-triggered PAR activation, short synthetic peptides derived from the proteolytically-exposed “tethered ligand” sequences are capable of PAR activation without receptor proteolysis [10,20] (Figure 1B). PAR_3_ appears to be the exception, where synthetic peptides corresponding to its thrombin-revealed sequence do not seem to cause PAR_3_ signalling [16] and instead are able to activate PAR_1_ and PAR_2_[21,22]. These so-called PAR-activating peptides (PAR-APs) have proved to be useful tools to study the function of PARs especially in settings in which more than one PAR subtype is expressed and stimulated by the same proteolytic enzyme [4,23]. Moreover, synthetic peptides derived from the ‘non-canonical’ cleavage of PAR_1_ (e.g. TLDPRSF-NH_2_ for a PR3 tethered ligand derived-activating peptide; or RNPNDKYEPF-NH_2_ for a NE tethered ligand-derived activating peptide) can serve as ‘biased’ agonists of PAR_1_ to activate MAPK but not calcium signalling [11]. These ‘biased signalling’ pathways that are selective for either G-protein-coupled responses or for beta-arrestin-mediated processes may lead to distinct receptor transactivation processes e.g. to release EGF-receptor transactivating ligands or prostaglandins that can in turn activate EP receptors (see below).

### PARs activate complex intracellular signalling networks

**Figure 2:**
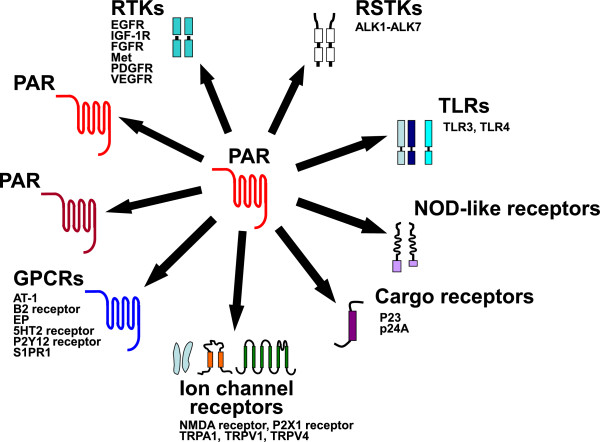
**PAR receptor crosstalk.** Scheme illustrating the interaction of PARs and their crosstalk with other receptors [GPCRs: G protein-coupled receptors (AT1: angiotensin receptor subtype 1, B2 receptor: bradykinin B2 receptor, EP: prostaglandin receptor, 5HT2 receptor: serotonin receptor subtype 2; P2Y12: purinergic ADP receptor; SP1PR1: sphingosine-1-phosphate receptor 1); PAR: proteinase-activated receptor; RTKs: receptor tyrosine kinases (EGFR: epidermal growth factor receptor; FGFR: fibroblast growth factor receptor; IGFR: insulin-like growth factor receptor; Met: hepatocyte growth factor (HGF) receptor; PDGFR: platelet derived growth factor receptor; VEGFR: vascular endothelial growth factor receptor); RSTKs: receptor serine/threonine kinases (ALK: activin-like kinase); TLRs: toll-like receptors (NLRs: NOD-like receptors, nucleotide oligomerization domain receptors); NMDA receptor: N-methyl-D-aspartate receptor; P2X1 receptor: ATP-gated cation channel; TRPA1: transient receptor potential ankyrin A1; TRPV: transient receptor potential vanilloid; p23, p24A: transmembrane proteins of the early secretory pathway. PARs can form homomeric interactions (indicated by light red-light red coloured symbols) or heteromeric interactions with other PARs (light red-dark red coloured symbols).

At present, PAR signalling is known to activate several major signal pathways. Firstly, the ‘classical pathway’ in which receptor activation causes signalling via heterotrimeric guanyl nucleotide-binding proteins (G proteins) and downstream targets; secondly, a beta-arrestin pathway of signalling involving ligand-regulated scaffolds; and thirdly, by the transactivation of a variety of receptors and other signalling constituents. This third possibility can include: (1) the rapid cellular release of agonists like prostaglandins or EGF-receptor (EGFR) ligands that can trigger non-PAR receptors by an autocrine or paracrine mechanism, (2) an intracellular kinase pathway (e.g. Src-family tyrosine kinase) that targets and activates a receptor like the one for EGF in an agonist-independent way and (3) a direct or indirect impact of the PARs on other signal mediators, either via GPCR-dimer formation or via transactivation of cell signalling constituents like ion channels or toll-like receptors (TLRs) (see Figure 2 and below). Thus, the ‘transactivation’ mechanisms in which the PARs participate can involve not only ‘growth factor’ receptors and G protein-coupled receptors, but also a diversity of other ‘signal generators’ (Figure 2). Given the complexity of the intracellular signalling networks, the ability of PARs to generate a ‘biased signal’ adds yet another layer of flexibility to the ways in which PAR activation can regulate cell and tissue behaviour.

#### G protein-mediated signalling by PARs

Like other GPCRs, the PARs signal via a variety of G proteins, including G_q_, G_i_ and G_12/13_ but not directly via G_s_[24,25]. For G protein-mediated signalling, the receptor acts as a ligand-triggered guanine nucleotide exchange factor, stimulating the exchange of GTP for GDP in the G_α_ subunit of the heterotrimeric G protein oligomer. This exchange enables the ‘release’ of the G_α_ subunit from its tight binding to the G_βγ_ dimer subunit. Each of the G protein moities (G_α_-GTP and G_βγ_) are then independently able to interact with downstream signalling effectors like phospholipase C (G_q_) or ion channels (G_βγ_). This ‘dual effector’ signalling, resulting in principle from the same PAR-activated G protein heterotrimer (e.g. G_q_G_βγ_), can converge for complex downstream signalling, for instance leading to NF-κB activation and intracellular adhesion molecule-1 (ICAM-1) transcription by the engagement of parallel G_q_/protein kinase C (PKC)- and G_i_/phosphatidylinositol 3-kinase (PI3K) pathways that converge [26,27]. Alternatively, as already indicated, via a ‘biased signalling’ process, PARs can be activated to affect selectively MAPK signalling via a G_12/13_-triggered process, without causing a G_q_-mediated calcium signalling event [28]. This kind of selective signalling can depend not only on the agonist *per se* [e.g. thrombin, neutrophil elastase, MMP1 or activated protein C (APC) for PAR_1_] but also upon the membrane environment in which a PAR is localized. For instance, triggering of PAR_1_ localized in the caveolae by APC can signal via set of downstream effectors that are distinct from those regulated when thrombin activates PAR_1_ in a non-caveolar environment [24].

#### Beta-arrestin-mediated signalling scaffolds

During the past few years it has become clear, that GPCRs, in addition to signalling via G proteins, are able to use another strategy to regulate intracellular signalling pathways. They direct the recruitment, activation, and scaffolding of cytoplasmic signalling complexes via two multifunctional adaptor and transducer molecules, beta-arrestins 1 and 2 [29-31]. Within the PAR family, this non-G protein mechanism involves the beta-arrestin-mediated internalization of PAR-beta-arrestin signalling scaffolds to regulate the activation of effector molecules like MAPK and PI3K as described for PARs 1 and 2 [28,31-35].

The coupling of the PARs to either the G proteins or beta-arrestins is driven by ligand-triggered changes of receptor conformation that for other GPCRs is thought to involve the putative transmembrane helices 3 and 6 of the receptor [36,37]. Of importance, different agonists are in principle capable of driving different conformational changes in the receptor to result in selective interactions with different downstream ‘effectors’. This principle is in keeping with the ‘floating’ or ‘mobile’ receptor model developed in the mid-1970s [38,39]. More recently, the paradigm has been ‘reinvented’ and expanded to encompass the concept of ‘biased receptor signalling’ or ‘functional selectivity’ as outlined in detail elsewhere [40,41].

### PAR-stimulated signalling cascades via receptor ‘transactivation’

**Figure 3:**
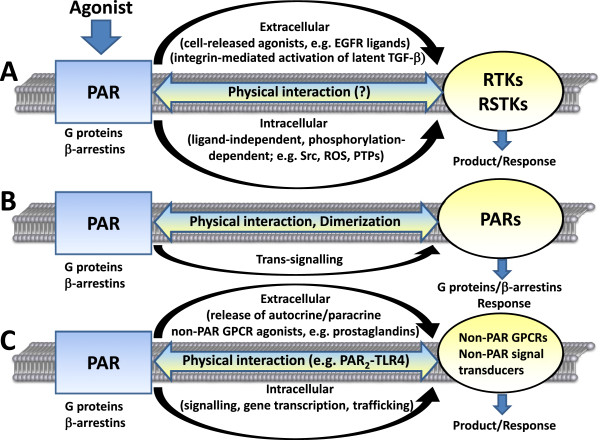
**Concepts and mechanisms of PAR receptor crosstalk with other receptors and signal transducers.** PAR receptor crosstalk involves **(A)** transactivation of receptor tyrosine kinases (RTKs) and receptor serine/threonine kinases (RSTKs), **(B)** PAR-PAR receptor interactions, and **(C)** PAR interplay with other non-PAR GPCRs and non-PAR signal transducers. **(A)** PARs can mediate transactivation of RTKs by an immediate matrix metalloproteinase (MMP)-catalysed release of RTK agonists from the cell surface, e.g. heparin-binding EGF, or transforming growth factor (TGF)-α, that in turn stimulates RTK signalling. PARs are also able to mediate transactivation of RSTKs by mechanisms including integrin-mediated activation of latent TGF-β. In addition, PARs can induce RTK transactivation via intracellular mechanisms including activation of Src, generation of reactive oxygen species (ROS), and inhibition of protein tyrosine phosphatases (PTPs). **(B)** PAR-PAR crosstalk involves PAR homo- and heterodimerization and PAR-PAR trans-signalling. **(C)** PARs are able to mediate transactivation of other non-GPCRs via extracellular release of GPCR agonists (e.g. the prostaglandin receptor by release of prostaglandins) and by intracellular mechanisms on the signalling (bradykinin B2 receptor, purinergic ADP receptor), gene transcription (angiotensin receptor subtype 1, serotonin receptor subtype 2), and receptor trafficking level. PARs further communicate with non-PAR signal transducers at both the signalling (toll-like receptors, ion channel receptors, NOD-like receptors) and receptor trafficking level (cargo receptors p23 and p24A).

The principle whereby an activated receptor can in turn, rapidly release a ligand that immediately ‘transactivates’ a downstream ‘receptor cascade’ is best illustrated by the agonist-driven release of nitric oxide, which immediately regulates tissue function. Although the ‘receptor’ for NO is an enzyme (guanylyl cyclase), its agonist-stimulated production immediately ‘transactivates’ downstream cellular signalling in a manner that reflects a receptor process. In this way, activation of PARs 1 and 2 in a blood vessel causes an immediate endothelium-dependent, NO-mediated relaxation of the tissue. In a similar way, PAR activation also causes the immediate production of prostaglandins, that in turn act in an autocrine or paracrine way to stimulate the prostanoid receptor (EP) family of GPCRs (see Figure 3). This prostaglandin-EP receptor transactivation rapidly affects vascular, airway and gastric smooth muscle relaxation. In this kind of situation, it is often a challenge to dissect the downstream signalling that is due either to the PAR or its co-ordinately transactivated ‘partner’ GPCR. Thus, for GPCRs, the term “transactivation” is taken to reflect “the activation of one GPCR that leads rapidly and in the absence of de novo protein synthesis to the activation and cytosolic generation of the immediate downstream signalling of a second cell surface receptor” [42]. This process is to be distinguished from a time-delayed PAR-mediated transcriptional-translational process (e.g. blocked by cycloheximide or actinomycin D), that over time (e.g. tens of minutes to hours) results in the secretion of agonists like cytokines.

Of importance, PARs seem also to be able to transactivate the sphingosine-1-phosphate receptor 1 (S1PR1) by a similar mechanism involving rapid release of its agonist, sphingosine-1-phosphate (S1P). This PAR-GPCR interplay was shown in endothelial cells [43,44] and in neural progenitor cells (NPCs) where an APC analogue stimulates neuronal function and differentiation via a PAR_1_-PAR_3_-S1PR1-Akt pathway. This result suggests the potential for APC-based clinical therapeutics for both development and repair in the human central nervous system [45].

#### Transactivation of receptor kinases via cell-released agonists

In addition to the immediate cascade-release of autocrine-paracrine agonists for GPCRs, it is now known that the activation of GPCRs, including the PARs, results in the cellular release of agonists that stimulate growth factor receptors like the one for EGF (see also Figure 3). Thus, activation of a GPCR results in an immediate matrix metalloproteinase (MMP)-catalysed release from the cell surface of an EGF-family EGFR agonist [heparin-binding EGF, or transforming growth factor-alpha (TGF-α)], that in turn activates receptor tyrosine kinase (RTK) signal pathways that are quite distinct from those activated by the GPCRs on their own [46-48]. This feed-forward signal cascade triggered by the receptor tyrosine kinase expands the range of the cellular functions attributable to PAR-mediated signalling networks. Thus far, this signalling paradigm has been described mainly for the EGFR, with little attention yet paid to a potential role for other RTKs like the receptors for hepatocyte growth factor (Met) and platelet derived growth factor (PDGFR). Nonetheless, since these initial findings, the ability of numerous GPCRs to transactivate RTKs has been found to involve not only the EGFR, but also the PDGFR, Met, the insulin-like growth factor receptor, (IGFR) and the fibroblast growth factor receptor (FGFR). In addition to the release of a cell-tethered EGFR ligand (e.g. heparin-binding-EGF; TGF-α) by matrix metalloproteinases (MMPs), the ‘transactivation process’ can also be attributed to (1) activation of Src-tyrosine kinase, (2) generation of reactive oxygen species (ROS) and (3) activation of protein tyrosine phosphatases (PTPs). All of these mechanisms are able to transfer signals indirectly from GPCRs to RTKs [49]. Importantly, recent data also point to the transactivation of receptor serine/threonine kinases (RSTKs) [42,50].

#### Transactivation of receptors via an intracellular mechanism

As already mentioned, in addition to its ‘transactivation’ by a cell membrane-released receptor agonist, data suggest that the EGFR can be activated in a ligand-independent way via an intracellular enzyme cascade involving Src-family kinase. Thus, in some circumstances, PAR-induced signalling cannot be blocked by a matrix metalloproteinase inhibitor (e.g. batimastat), but is diminished both by an EGFR-kinase-selective inhibitor (AG 1478) and by a Src-family-selective inhibitor (PP1). Therefore, it appears that a PAR-stimulated activation of Src leads via an intracellular route to a ligand-independent phosphorylation-dependent activation of the EGFR. Collectively, these data suggest a role for both kinds of ‘cascade’ receptor transactivation caused by PARs in several diseases including inflammation, cardiac injury, neurodegeneration and cancer (see Figure 3).

## Selected examples of PAR-stimulated receptor transactivation

Transactivation of other receptors by PAR activation, as outlined above, is now known to be a common phenomenon. In the following sections, we describe some selected illustrative examples of PAR-stimulated receptor transactivation to provide a perspective on the versatility of this kind of signalling process. These illustrations are indicative of many other examples to be found in the literature. Further, we deal with the potential impact of PAR-triggered receptor transactivation in both normal and pathophysiological settings. In that context, we discuss the potential involvement of the PARs and their transactivation mechanisms in the pathophysiology of vascular disease, inflammation and cancer.

### A transactivation signalling network between PAR_1_, the epidermal growth factor receptor (EGFR) and the vascular endothelial growth factor receptor (VEGFR)

In a study on endothelial cells (ECs) Chandrasekharan and colleagues provided an interesting example for a complex metalloproteinase-independent, but EGF-dependent signalling interaction between PAR_1_, the EGFR and the VEGFR, resulting in transcriptional activation by mitogen-activated protein kinase phosphatase 1 (MKP-1), a key signalling mediator in thrombin and VEGF-mediated activation of endothelial cells. This signalling interplay network uses both p42/p44 MAPK-dependent and p42/p44 MAPK-independent pathways, the latter of which involve c-Jun N-terminal kinase (JNK) activity and the VEGFR-2 [51]. This report is particularly important since it demonstrates for the first time interactions between a GPCR, the EGFR, and the VEGFR leading to gene activation on a transcriptional level. Moreover, it underlines the significance of this complex receptor interplay in the vascular microenvironment.

### PAR-mediated transactivation of platelet-derived growth factor receptor (PDGFR), Met and insulin-like growth factor-1(IGF-1) receptor

In contrast with numerous studies providing evidence for a crosstalk between PARs and the EGFR, there is only very limited information about PAR-mediated transactivation of other receptor tyrosine kinases.

Siegbahn et al. demonstrated that the tissue factor-factor VIIa (TF/FVIIa) complex is able to transactivate PDGFR-β [52]. Since TF/FVIIa is known to be able to activate PAR_2_, this ability of TF/FVIIa to activate the PDGFR-β may be due initially to PAR_2_ activation. Further evidence for this PAR_2_-PDGFR crosstalk comes from the observation that the PAR_2_-selective agonist peptide, 2-furoyl-LIGRLO-NH_2_, induces phosphorylation and activation of the PDGFR in liver carcinoma cells [53]. Since the PAR_1_-selective agonist peptide, TFLLRN-NH_2_, and the PAR_4_-selective agonist peptide, AYPGKV-NH_2_, can also induce activation of the PDGFR in Hep3B liver carcinoma cells, a coordinated receptor tyrosine kinase signalling of the PARs 1, 2 and 4 in liver carcinoma cells may be suggested [53].

In addition to causing a transactivation of the PDGFR, PAR_2_ stimulation leads to a transactivation of Met. In liver carcinoma cells, this PAR_2_-triggered transactivation of Met promotes cell migration and invasion [54,55].

Finally, PAR_1_ has been reported to mediate transactivation of the IGF-1 receptor by a mechanism that is involved in the regulation of aortic smooth muscle cell proliferation [56-58]. In sum, the above-cited examples show that PAR activation can result in the transactivation of a variety of receptor tyrosine kinases in addition to activating the EGF receptor.

### PAR-mediated receptor-serine/threonine- kinase (RSTK) transactivation

So far, the model of receptor transactivation by PARs has dealt primarily with the receptor tyrosine kinases discussed in the previous sections. However, recent data suggest that this signalling paradigm can be extended to include receptors with intrinsic serine/threonine kinase activities (RSTKs) [42,50,59] (see Figure 3). Specifically, it is now evident that GPCR agonists can transactivate the TGF-β/activin/BMP superfamily of growth factor receptors, all of which possess serine/threonine kinase activity and signal through SMAD proteins (for review see e.g. [60]). The extension of GPCR transactivation to include RSTKs provides for a new spectrum of cellular responses that PARs can stimulate, downstream of the canonical SMAD signalling pathway. An interesting example can be found in the ability of thrombin in mouse lung epithelial cells [61] and vascular smooth muscle cells [59], to cause a transient increase in C-terminally phosphorylated SMAD2 levels (pSMAD2C). In the latter cells, the pSMAD2C phosphorylation can be blocked by the PAR_1_ antagonists JNJ5177094 and SCH79797, as well as by SB431542, an inhibitor of the TGF-β type I receptor ALK5 [59]. Sensitivity to SB431542 confirms that the SMAD2C phosphorylation arises directly from the serine/threonine kinase activity of ALK5 and indicates that agonist stimulation of PAR_1_ can mediate transactivation of ALK5. Interestingly, in mouse lung epithelial cells the transactivation mechanism involves an αVβ6 integrin-Rho/rho kinase (ROCK) signalling link to RSTK activation [50]. The mechanism of PAR_1_ transactivation of ALK5 matches the extracellular, ligand-dependent type of transactivation, involving binding and activation of released latent TGF-β. Ligation and activation of PAR_1_ causes αVβ6 integrin activation via RhoA/ROCK [62] and integrin binding to the Large Latent Complex causes its conformational change resulting in exposure of the TGF-β dimeric ligand [61]. The PAR_1_-mediated enhancement of αVβ6-dependent TGF-β activation finally results in activation of the ALK5 kinase. Through an overstimulation of this ligand-dependent mechanism, PAR_1_ is capable of promoting acute lung injury [61]. In contrast, the generation of pSMAD2C in vascular smooth muscle cells in response to thrombin treatment is not due to the agonist-mediated release and autocrine action of TGF-β since the generation of pSMAD2C could not be blocked by a neutralising pan TGF-β antibody [59]. Thus, this transactivation event is ligand-independent and appears to be of the intracellular type although the precise mechanism is not known at present.

We speculate that ALK5 will not remain the only receptor from the TGF-β/activin/BMP superfamily of growth factor receptors that is a target of transactivation by PARs. Given the high homology among the ALK5 subgroup of TGF-β type I receptors, encompassing ALK5, and the activin receptors ALK4 and ALK7 (which is reflected in the fact that they share sensitivity to SB431542), it is likely that PAR ligands will also display activin-like effects through transactivation of ALK4 and/or ALK7 and thus participate in classical activin responses like stimulating the proper development of the endocrine and exocrine pancreas [63]. If we take this speculation further, transactivation by PARs of the BMP receptors ALK1, ALK2, ALK3 (BMPR1A), and ALK6 (BMPR1B) might enable PARs to stimulate phosphorylation of SMADs 1, 5 and 8 and hence a plethora of BMP-specific biological responses. A precedent for such an interaction is the GPCR agonist serotonin, which in pulmonary arterial smooth muscle cells stimulates an increase in serine/threonine phosphorylation of ALK3, thereby leading to the phosphorylation of SMADs 1, 5 and 8 and their subsequent nuclear translocation [64].

Our own results (H. U., F. G., unpublished observations) have shown that in some tumor cell types, PAR_2_ expression is required for efficient TGF-β/ALK5-mediated SMAD3C phosphorylation and for certain TGF-β-stimulated responses, such as cell migration. We are currently studying whether a ‘reverse’ transactivation (from ALK5 to PAR_2_) can occur. That process would enable TGF-β to signal via PLC, with the generation of InsP_3_ and diacylglycerol. In rat astrocytes, TGF-β stimulation has indeed been shown to result in a GPCR-mediated activation of PLC [65]. The rapid TGF-β-mediated release of a GPCR agonist like a prostaglandin, as discussed in the next section, might be involved in this kind of reciprocal TGF-β-GPCR transactivation process. That possibility has yet to be explored in depth.

### PAR transactivation of prostanoid receptors

Besides its ability to induce pro-inflammatory effects [66], an anti-inflammatory role of PAR_2_ in the airway has also been described [67,68] in accordance with the ability of PAR_2_ activation to cause the secretion of prostaglandin E2 (PGE2) from the airway epithelium [67,69-72]. The released prostanoids can cause anti-inflammatory effects mainly through the activation of the prostanoid receptor (EP) subtypes EP2, EP3 and EP4 [70,73-77]. The PAR_2_ interplay with PGE2/EP-signalling in the airway system, defined as a PAR_2_-prostaglandin E2-prostanoid EP receptor axis [78], involves a signalling network triggering arachidonic acid release by the p42/p44 MAPK/cytosolic phospholipase A2 (cPLA2)-pathway downstream from PKC and non-Src tyrosine kinases, upregulation of COX-2 via Src/EGFR/p38 MAPK, and cyclooxygenase-2 (COX-2)-independent NF-κB signalling [69,79-81]. Using HEK 293 T cells, Komatsu et al. provided a novel mechanistic aspect for a PAR_2_-PGE2/xEP2 interplay which points to a PGE2-initiated inhibition of PAR_2_-dependent signal transduction by inducing PAR_2_ internalization through a prostanoid receptor subtype EP2-mediated increase in intracellular cyclic AMP [82]. Interestingly, for PAR_1_ which is also known to be able to induce PGE2 secretion from human respiratory epithelial cells [83] and peritoneal macrophages [84], a very similar mechanism has been described in lung fibroblasts [85].

## PAR-triggered receptor transactivation: pathophysiological implications

In addition to the above mentioned work by the Ullrich group [46], further studies demonstrating the ability of thrombin and its precursor enzyme, prothrombin, to induce EGFR activation [46,86-88] points to the participation of PARs in this “RTK transactivation” pathway in many physiological settings. Following are some selected examples of this transactivation that has a potential impact on several cellular processes. The examples are not meant to be comprehensive, but rather illustrative of several pathophysiological settings in which PAR-receptor kinase transactivation can play a role.

### PAR-mediated RTK transactivation and the cardiovascular system

Over the past decade work has mainly been focused on the ability of the PARs to transactivate the EGFR. In 2002, Sabri et al. showed that epidermal growth factor-like EGFR transactivation is involved in PAR_1_-triggered stimulation of p42/p44 MAPK that results in cardiac fibroblast proliferation [89]. Interestingly, further research in this field on mouse cardiomyocytes demonstrated that PAR_4_ is also able to transactivate the EGFR and its related family member, ErbB-2, by a mechanism involving Src tyrosine kinase and both p42/p44 MAPK and p38 MAPK [90]. Thus, PAR_1_ and PAR_4_ signalling is predicted to contribute to remodeling during cardiac injury and/or inflammation via this transactivation mechanism. Further, both PAR_1_- and PAR_4_-mediated EGFR transactivation signals are thought to be involved in the regulation of cardiac physiological and pathophysiological functions.

In addition to the tissue kallikrein (TK)/kallikrein-related peptidase (KLK) family, a distinct plasma kallikrein (PK) family member has been shown to activate PAR_1_ and PAR_2_ in primary rat aortic vascular smooth muscle cells [91]. This activation sequentially leads to the metalloproteinase (ADAM17)-triggered release of the EGFR agonist, amphiregulin and tumor necrosis factor-alpha (TNF-α). Amphiregulin and TNF-α, via their respective receptors (EGFR, TNFR), result in the activation of p42/p44 MAPK [91]. These data indicate that two distinct ‘kallikrein’ families (KLKs and PKs) may contribute to the regulation of vascular responses in pathophysiologic states.

Al-Ani and colleagues showed that endothelial PAR_2_ mediates enhanced expression and release of soluble VEGF receptor-1 (sVEGFR-1) in cultured human umbilical vein endothelial cells (HUVECs) from preeclamptic pregnancies. This mechanism involves PKC-driven transactivation of the EGFR. This process might be relevant for preeclampsia which is characterized by widespread maternal endothelial damage and occurs as a consequence of elevated sVEGFR-1 in the maternal circulation [92].

### PARs, epidermal growth factor receptor transactivation and the skin

Recent studies have shown that members of the tissue kallikrein (TK) or kallikrein-related peptidase (KLK) gene family can play diverse roles in regulating peripheral tissue inflammation, repair and pain by activating PAR_1_, PAR_2_ and PAR_4_[93,94]. Based on findings that the shedding of EGFR ligands is required for keratinocyte migration in cutaneous wound healing [95], Gao et al. demonstrated a novel signalling pathway mediated by tissue kallikrein-KLK1 via PAR_1_ and EGFR activation in the migration of cultured HaCaT keratinocytes; and they provided evidence for the significance of this mechanism in vivo using a skin wound healing model in rats [96]. This pathway includes PAR_1_-mediated PKC-Src signalling and EGFR transactivation, resulting from the MMP-catalyzed release of the EGFR-activating ligands, heparin-binding-EGF (HB-EGF) and amphiregulin.

### PAR-mediated receptor tyrosine kinase transactivation in arthritis, inflammation, and pain

Thrombin is known to be involved in the regulation of fibrin deposition, angiogenesis, cell invasion and proinflammatory processes. Abnormalities in these inflammatory events are primary features of both rheumatoid arthritis and osteoarthritis. Recently, Huang and colleagues demonstrated the involvement of PAR_1_-mediated Src-dependent EGFR transactivation in the thrombin-induced expression of chemokine (C-C motif) ligand-2 (CCL2) in human osteoblasts [97]. Since CCL2 is well known to be implicated in rheumatoid arthritis [98], a role for a PAR_1_-EGFR transactivation interplay in this inflammatory disease has been suggested. Further, both PARs 2 and 4 have been implicated in arthritis pain as well as inflammation [99-102]. In an adjuvant model of arthritis, PAR_2_ has been found to play a critical role [103], but the precise mechanisms whereby PAR_2_ promotes joint inflammation, possibly involving RTK transactivation are not yet known.

### PARs, receptor tyrosine kinase transactivation and the respiratory system

In the respiratory system, PAR_1_, PAR_2_ and PAR_4_ are expressed at different levels depending on the tissues or the cell types (epithelium, endothelium, tracheal smooth muscle and blood vessel), and contribute to the progression of various airway and lung disorders including inflammation and fibrosis [23,104,105]. Activation of PAR_2_ in particular by allergen-derived proteinases is believed to contribute to lung tissue eosinophil influx [106,107]. However, the signal pathways that involve both beta-arrestin-mediated and beta-arrestin-independent mechanisms for allergen proteinase-induced lung inflammation have yet to be determined. Whether EGFR transactivation is involved has not been evaluated. Recently, Ando et al. demonstrated that PAR_4_-mediated EGFR signalling plays a role in alveolar epithelial-mesenchymal transition (EMT), an important mechanism in pulmonary lung fibrosis [108]. In addition, EGFR activation has been found to be involved in PAR_2_-triggered signal transduction pathways that contribute to a post-transcriptional process for the release of IL-8 in human lung epithelial cells [109]. Thus, PAR activation with or without a transactivation of the EGFR is of importance in the pathophysiology of the lung.

### PARs, receptor tyrosine kinase transactivation and cancer

It is now widely accepted that EGFR transactivation in response to the stimulation of GPCRs occurs in a large number of cancer cells, and it is believed that this mechanism is an important signalling principle contributing to cancer development and progression [110]. For example, there is a growing body of literature describing the ability of PAR_1_ and PAR_2_ to transactivate the EGFR in cells from several carcinomas including lung [69], kidney [111], colon [112-115] and gastric cancer [116,117]. A substantial amount of data point to an important role for PARs in colon cancer. In cells from this tumor entity, PAR_1_ and PAR_2_ have been demonstrated to induce migratory and proliferative effects that involve both activation of p42/p44 MAPK and transactivation of the EGFR [112-114]. In addition, PAR_4_ has recently surfaced as a new important player in the regulation of colon tumor-derived cells. In colon carcinoma cells activation of PAR_4_ has been found to be involved in stimulating mitogenesis. This stimulation is observed to occur in the setting of PAR_4_-induced increases in intracellular calcium and activation of p42/p44 MAPK along with transactivation of ErbB-2, but not via crosstalk with the EGFR [118]. In this setting, the localized selective induction of KLK14 in the colon cancer cells, but not in adjacent uninvolved colon epithelium may play an important role by cleaving and activating PARs [119,120].

In renal carcinoma cells, the matrix metalloproteinase (MMP) inhibitor GM 6001 diminishes the tyrosine phosphorylation of the EGFR induced by PAR_1_, pointing to a critical involvement of metalloproteinase activity in the PAR_1_-mediated transactivation of the EGFR in renal carcinoma cells [111]. A similar mechanism, with the participation of MMPs, has been shown in colon carcinoma cells where PAR_1_-mediated enhanced cell proliferation is stimulated by an MMP-dependent transactivation of the EGFR [121]. As alluded to above, in a separate cell system (cardiac fibroblasts), PAR_1_ activation results in EGFR trans-phosphorylation in an MMP-independent Src family kinase-dependent process [89]. Those distinct results imply that PAR_1_-mediated EGFR transactivation signalling is contextual in nature, depending on the cell type in which the EGFR and PAR_1_ reside.

Arora et al. showed that proteolytic activation of PAR_1_ by thrombin induces persistent EGFR and ErbB-2 transactivation in invasive breast cancer cells. This result is distinct from the transient EGFR and ErbB-2 transactivation observed in normal mammary epithelial cells. In addition, these authors demonstrated that PAR_1_-stimulated EGFR and ErbB-2 transactivation sustains p42/p44 MAPK signalling and promotes breast carcinoma cell invasion [122].

Besides a role for PARs in growth and metastasis formation in carcinoma, there is growing evidence that chronic inflammation, resulting in increased pro-inflammatory mediators like prostaglandins produced by up-regulated cyclooxygenase (COX) plays a role in neoplastic transformation [123,124]. In this regard, PAR_2_ signalling is known to be critically involved in inflammatory processes in different organs including the gastrointestinal system [125,126]. Thus, by increasing prostaglandin production, crosstalk of PAR_2_ with PGE2/EP signalling may be involved in the progression from chronic inflammation to cancer in the intestine. A PAR_2_-triggered transactivation of the EGFR appears to be involved in this PAR_2_-driven process. This possibility is illustrated by a study in intestinal epithelial cell-6 cells (IE6) and Caco-2 colon cancer cells in which PAR_2_-driven prostaglandin E-2 (PGE2) production is a consequence of increased COX-2 expression, that results from a metalloproteinase-dependent transactivation of the EGFR. This process leading to COX-2 upregulation and an increase in prostaglandin production results from the activation of Src, Rho, and PI3K signalling [127].

## Receptor-receptor interactions – a critical element in PAR signal transduction

In addition to the ability of PARs to transactivate other GPCRs, like the EP family, and receptor-kinases like the EGFR, it is now accepted that receptor-receptor GPCR dimer formation plays an important role in both physiological and pathophysiological settings. For the PARs, these dimers include PAR-PAR homo- and heterodimers, as well as PAR interactions with other G-protein coupled receptors (bradykinin receptor, prostanoid receptor, P2Y receptor, alpha adrenergic receptor, serotonin receptor and angiotensin AT1 receptor). Direct or indirect PAR interactions with toll-like receptors (TLRs) and NOD-like receptors (NLRs) to generate signal crosstalk are also of importance. Furthermore, PAR signalling is now known to involve crosstalk between PARs and multi-subunit ion channel receptors (NMDA receptor, P2X1 receptor), transient-receptor-potential channels (TRPV1, TRPV4 and TRPA1)], and cargo receptors (p23, p24A) (see Figures 2 and 3). These mechanisms whereby PARs can ‘crosstalk’ via direct and indirect interactions with other GPCRs and with other signal-generating targets add substantial signalling complexity over and above the ways in which PARs can regulate cell function by transactivating receptor-kinases. The following sections deal with these types of PAR-PAR and PAR-non-PAR interactions.

### PAR-PAR interactions – a role for receptor dimer formation

Since the mid-1990s a growing body of pharmacological, biochemical and biophysical data indicate that GPCRs form functional homo- and heterodimeric complexes. It is now widely accepted that dimerization is a universal aspect of GPCR biology [128-131]. GPCR dimerization involves the formation of functional physical ‘pairs’ of receptor units (homo- or hetero-partners). This process leads to an increase in the diversity of receptor function, since the ‘dimers’ can interact with an expanded spectrum of downstream signal transducer elements, as foreseen by the floating or mobile receptor hypothesis outlined some time ago and recently updated [38-41,132,133]. This concept also includes the potential for GPCRs to interact directly with several different non-GPCR signalling proteins like the toll-like receptors (see below) to generate complex downstream signals and is emerging as increasingly important in creating functional receptor diversity [134].

In principle for the PARs, all of PARs 1, 2, 3 and 4 can synergize for signalling and can potentially form PAR homo- or heterodimers. During the past few years PAR-PAR interactions have been studied and several models of PAR trans- and coactivation have been proposed in different cell types, suggesting a role for PAR-PAR physical association [16,135-142]. However, only limited data exist about PAR homo- and heterodimer complex formation and their signalling impact in these cells; and most of the work has been done with expression systems in which higher than normal receptor concentrations may drive PAR-PAR dimer formation in a way that does not operate in naturally occurring cells. For instance, the platelet represents one of the few systems in which PAR_1_-PAR_4_ heterodimerization has been evaluated directly in the setting of endogenous PAR expression [143]. Otherwise, as for GPCRs in general [144], the “dimer field” has been dominated by techniques involving recombinant cell lines expressing mutant receptors, often involving the solubilization of the receptors. The techniques used for monitoring homo- and heterodimer formation by GPCRs, including fluorescence resonance energy transfer (FRET) or bioluminescence resonance energy transfer (BRET) are a challenge for use in studying the low abundance of receptors in many cells endogenously expressing PARs, with the added complexity of background fluorescence [145,146]. For that reason, the PAR-PAR dimerization models obtained from cell expression systems illustrate the oligomerization that is indeed possible, but may not necessarily reflect a physiological state for tissues *in vivo*. These studies using fluorescence/bioluminescence emission (FRET/BRET) and biochemical approaches (immunoprecipitation-gel electrophoresis-western blot) can be complemented by methods using time-resolved fluorescent energy transfer (TR-FRET) involving Snap-tag chemistry to allow for the direct identification of wild-type GPCR dimerization *in vivo*[147,148]. With the above caveat for interpreting data obtained using receptor expression systems, the following sections summarize the potential PAR-PAR interactions that may govern their signalling properties.

#### Evidence for PAR-PAR proximity and signalling ‘in-trans’ by a proteinase-revealed tethered ligand

The first indication that PARs can interact synergistically for signalling came from studies of the PAR_1_ tethered ligand signalling mechanism [136]. In that study, it was found that PAR_1_ lacking its ‘tethered ligand (TL) sequence’ could be activated by the action of thrombin to reveal a ‘tethered ligand agonist’ on a ‘partner’ PAR_1_ that had an intact tethered ligand sequence, but was not able to signal on its own [136]. This work was followed some time later by studies showing that in cultured human umbilical vein endothelial cells, the tethered ligand of cleaved PAR_1_ can ‘reach over’ to transactivate PAR_2_[135]. These results were obtained at the time when it was already known that PAR_3_ can act to sensitize PAR_4_ for thrombin action, implying a proximity of PARs 3 and 4. However, direct PAR-PAR interactions determined by physicochemical methods had not yet been documented. The following sections deal with evidence for the formation of physical PAR-PAR dimers.

#### PAR_1_-PAR_2_ dimerization

The work pointing to PAR-PAR interactions summarized in the preceding paragraph was followed by more direct measurements of PAR-PAR signalling crosstalk and interactions. Signalling crosstalk between endothelial PAR_1_ and PAR_2_*in vivo* has been demonstrated in a sepsis mouse model, where the protective effect of PAR_1_ agonist activity in endothelial barrier function and survival in mice required the presence of PAR_2_[149]. On a signal transduction level, PAR_1_ was found to couple to G_12/13_-Rho pathways while PAR_2_ coupled to a G_i_-Rac signalling route in human pulmonary artery endothelial cells (HPAECs). Therefore, in terms of signalling, PAR_2_ appeared to dominate over PAR_1_ and transactivation of PAR_2_ by PAR_1_ promoted barrier-protective G_i_-Rac signalling. Since FRET studies detected PAR_1_ and PAR_2_ in close molecular proximity in cytoplasmic vesicles and on the plasma membrane in cells from the permanent endothelial cell line EA.hy926 [149], it can be suggested that PAR_1_-PAR_2_ heterodimer formation is involved in the transactivation of PAR_2_ by PAR_1_, switching the physiological response of the endothelial cells from barrier disruptive to barrier protective. Transactivation of PAR_2_ by thrombin-cleaved PAR_1_, that has also been demonstrated on human umbilical vein endothelial cells (HUVECs) [135], underlines the potential function of PAR_1_ heterodimer formation with PAR_2_ in endothelial cells.

In addition, physical association and functional coupling between PAR_1_ and PAR_2_ on vascular smooth muscle cells (VSMCs) seems to be responsible for the ability of PAR_2_ to regulate the PAR_1_ hyperplastic response to arterial injury leading to stenosis [150]. Thus, in several settings in the vasculature, PAR_1_-PAR_2_ heterodimers may be of relevance for signalling and the development of PAR antagonists will need to take this aspect into account.

In addition to the vascular system, cooperative signalling between PAR_1_ and PAR_2_ has been observed on carcinoma cells and therefore suggests a role of PAR_1_-PAR_2_ dimerization in carcinogenesis. For example, studies on melanoma cells have indicated that stimulation of cell motility by thrombin requires not only the activation of PAR_1_ but also the simultaneous activation of PAR_2_[137]. In breast carcinoma cells PAR_1_-PAR_2_ receptor complexes seem to reside in different membrane microdomains since thrombin but not factor Xa activated the PAR_1_-PAR_2_ response in breast cancer cells [141]. This impact of PAR location in the caveolar domain has been pointed out for the endothelial activation of PAR_1_ by activated protein C (APC), to drive signalling in a direction very distinct from that triggered by thrombin in platelets [151]. Whether PAR_1_-PAR_2_ dimer formation is an issue for APC signalling remains to be determined. An intriguing impact of PAR_1_-PAR_2_ heterodimer formation on signalling has come from work in the Trejo laboratory [152] indicating that PAR_1_ and PAR_2_ form a heterodimer that exhibits unique trafficking and signalling behaviours compared with receptor protomers. Strikingly, this study showed that thrombin-activated PAR_1_/PAR_2_ heterodimers signal via a beta-arrestin-scaffold-mediated activation of MAPK in the cytoplasm, whereas the activation of the PAR_1_ monomer by thrombin promotes its redistribution to the nucleus, presumably for a signalling function. Thus, in targeting the PARs for cancer therapy, PAR_1_/PAR_2_ dimer formation will also prove to be a factor.

#### PAR_1_-PAR_4_ dimerization

The cooperative PAR_1_/PAR_4_ receptor system which has been described [153-155], indicates that both receptors cooperate to mediate human platelet signalling and aggregation at both low and high thrombin concentrations, respectively. However, those first studies did not document a physical interaction between PARs 1 and 4, although the data unequivocally pointed to such interactions. Using different sophisticated western blotting and co-immunoprecipitation approaches, Kuliopulos and coworkers demonstrated that PAR_1_ and PAR_4_ associate as a stable heterodimeric complex in human platelets. The data obtained provide evidence for a mechanism by which thrombin first docks to and cleaves PAR_1_, and then reaches over and cleaves PAR_4_ while still bound to PAR_1_[143]. Therefore, it has been concluded that PAR_1_-PAR_4_ dimerization enables thrombin to function as a bivalent agonist. This mechanism might contribute to the biphasic kinetics of activation and signalling for PAR_1_ and PAR_4_ by thrombin in human platelets [156,157]. This PAR-PAR interaction concept was supported further by co-immunoprecipitation and FRET studies demonstrating the ability of PAR_1_ and PAR_4_ to form heterodimers in COS-7 fibroblasts transfected with PAR_1_ and PAR_4_[143].

There are also results suggesting the formation of PAR_1_-PAR_4_ heterodimers in other cell types including those from epithelial cancers. For instance, a PAR_1_-PAR_4_ two-receptor system has been demonstrated to mediate a closely related thrombin-induced signalling in both astrocytoma [138] and hepatocellular carcinoma [158] cells where PAR_1_ and PAR_4_ clusters could be detected by a high-resolution field emission scanning electron microscopy (FESEM) freeze-fracture replica immunolabeling technique. Although not accepted in general as a method to verify receptor dimerization, these data indicate structural proximity of PAR_1_ and PAR_4_ and therefore underline the need to evaluate the PAR dimerization concept in future studies of neoplastic cells.

#### PAR_1_-PAR_3_ dimerization

In contrast with the situation found in human platelets, murine platelets lack PAR_1_ and instead express a high-affinity thrombin-binding receptor, PAR_3_, in addition to PAR_4_ which binds thrombin with lower affinity [153]. As already alluded to above, upon cleavage by thrombin, PAR_3_, rather than itself mediating intracellular signalling, functions as a cofactor facilitating thrombin-induced activation of PAR_4_[16,159]. In contrast with the characterization of PAR dimerization in human platelets, there are as yet no conclusive data establishing a direct physical association between PAR_3_ and PAR_4_. However, receptor dimerization in platelets is likely since X-ray crystallographic studies show that the synthetic peptides representing the thrombin-target tethered ligand sequences of PARs 3 and 4 can bind to thrombin in a way that would enable a ‘crosslinking’ of both PARs 3 and 4 by interactions with thrombin’s exosite [159]. The consequence of such a thrombin-linked ternary complex where the receptors can interact in terms of signalling remains to be determined [16,159].

All four members of the PAR family are expressed in arterial and/or venous endothelial cells [160-164]. Therefore, these cells are potentially very interesting for studies on receptor dimer formation. Using human pulmonary artery endothelial cells (PAECs) and HEK 293 T cells, McLaughlin et al. were able to detect heterodimer complexes using BRET-2 [165]. They found that PAR_3_ directly dimerizes with PAR_1_ to induce a specific PAR_1_/G_13_-binding conformation that favors G_13_ activation. From these results the authors propose a model of PAR_1_ activation involving the interaction of PAR_1_ with PAR_3_, which alters the selectivity of PAR_1_ for G_13_ coupling and finally promotes endothelial barrier dysfunction. Therefore, PAR_3_ seems to function as an allosteric modulator of PAR_1_ signalling through dimerization with PAR_1_ in endothelial cells and to favor a distinct G_13_-activated downstream signalling pathway.

#### PAR_2_-PAR_4_ dimerization

Very recently, PAR_2_-PAR_4_ heterodimer formation was detected in transfected keratinocyte NCT-2544 cells and in human embryonal kidney HEK 293 T cells using FRET and co-immunoprecipitation techniques. This dimerization was shown to play a role in membrane trafficking and signal transduction of PAR_4_ in these cells [166]. This study provides the first evidence for a functional PAR-PAR interaction where PAR_2_-PAR_4_ hetereodimer formation is facilitated by the plasma membrane delivery of PAR_4_ through disruption of its binding to the endoplasmic reticulum protein, COP1 β-subunit (β-COP1), and by the interactions of PAR_4_ with the chaperone protein 14-3-3ζ. Of note, the association of PAR_2_ with PAR_4_ markedly enhanced PAR_4_-mediated ^3^H inositol trisphosphate (InsP3) accumulation in NCT-2544 cells [166].

#### PAR_4_-PAR_4_ homodimerization

In addition to heterodimerization there are now data demonstrating PAR-PAR homodimer complex formation. Using bimolecular fluorescence complementation (BiFC) and BRET, de la Fuente and colleagues provided evidence for PAR_4_ homodimer complexes in HEK 293 T cells transiently transfected with PAR_4_[167]. Using a panel of chimeric proteins and PAR_4_ point mutants the authors were able to map the region on PAR_4_ required for homodimers to a hydrophobic interface within transmembrane helix 4. In addition, they showed that point-mutations that disrupt PAR_4_ homodimers also impair signalling as measured by calcium mobilization [167]. As outlined above, PAR_4_ may form heterodimer complexes with PAR_1_[143,153-157] and PAR_2_[166], respectively. In this context, it will be interesting to investigate the impact of PAR_4_ homodimerization in relation to the physical association of PAR_4_ with the other members of the PAR family, namely, PAR_1_ and PAR_2_. One aspect of the PAR-PAR homo- or heterodimer function that has yet to be evaluated relates to the ‘biased’ signalling properties of PARs which are activated at ‘non-canonical’ cleavage sites to generate diverse ‘tethered ligand’ agonists. Since these different ‘tethered ligands’ will confer distinct active receptor conformations, it is likely that the function of any putative PAR-PAR dimeric species will differ considerably, depending on the sequence of the proteinase-revealed tethered ligand. This issue related to ‘biased signaling’ by unique tethered ligands or by different PAR biased agonists/antagonists has not yet been evaluated and merits further attention.

### PAR crosstalk with other signal transducers including non-PAR G protein-coupled receptors, toll-like receptors, ion channel receptors, transient receptor potential ion channels, NOD-like receptors and cargo receptors

As already discussed briefly above, two different mechanisms are critically involved in PAR receptor crosstalk: (1) receptor transactivation and (2) receptor dimerization/oligomerization. However, PARs are also capable of communicating with various types of non-PAR signal ‘transducers’ , including other GPCRs (P2Y12 receptor, bradykinin B2 receptor, 5HT2 receptor, angiotensin AT1 receptor), TLRs, ion channel receptors, transient receptor potential ion channels, NOD-like receptors and cargo receptors. In the following sections, we will provide an overview dealing with the crosstalk between PARs and those other non-PAR signal transducers. Since for PARs no data have yet been published about physical interactions with other signal transducers, including the other GPCRs, the following sections are focused on the interplay of PARs with different signalling elements via their signal transduction pathways, including interactions at the level of gene transcription.

### Crosstalk on a receptor signalling pathway level

#### Other G protein-coupled receptors

##### Interaction of PAR_1_/PAR_4_ with purinergic P2Y receptor subtype, P2Y12

In human platelets, the purinergic P2Y12 receptor promotes thrombin- and collagen-induced procoagulant activity [168] and induces the generation of the lipid mediator, thromboxane A2 (TXA2) [169]. This increase in TXA2, known to be mediated by activation of PAR_1_ and PAR_4_[170-172], serves to recruit other platelets to the site of injury and reinforces the platelet plug. The coordinated action of PARs 1 and 4, along with the purinergic P2Y12 receptor to cause TXA2 generation has been investigated in more detail. According to a working model [169] activation of phospholipase C-β (PLCβ), results in an inositol (1,4,5) trisphosphate-stimulated release of calcium from intracellular stores and an activation of protein kinase Cs (PKCs). PLCβ activation and elevated intracellular calcium are critical for the downstream activation of Src kinase, which then induces p42/p44 MAPK activation. Both elevated intracellular calcium and activation of PKCs lead to the secretion of adenosine diphosphate (ADP) from the platelet-dense granules and an initiation the primary phase of thromboxane A2 (TXA2) generation. In a secondary phase, the secreted ADP activates the G_i_-coupled P2Y12 receptor leading to a potentiation of the PAR-mediated activation of p42/p44 MAPK and TXA2 generation [169]. Furthermore, Li and colleagues demonstrated on human platelets a direct interaction of the P2Y12 receptor with PAR_4_ which regulates arrestin recruitment of PAR_4_ and is thought to contribute to thrombus formation in vivo [173]. Thus, it appears that PAR-P2Y12 interactions occur for PAR_4_ and may take place for other PARs.

##### Interaction of PAR_4_ with the bradykinin B2 receptor

Recent evidence suggests that the pro-inflammatory effects of PAR_4_ activation reported frequently by several groups [174-176] are dependent on signalling by the bradykinin B2 receptor (B2 receptor), since oedema in a rat paw inflammation model induced by the PAR_4_-selective agonist peptide AYPGKF-NH_2_ can be blocked by administration of the B2 receptor antagonist, HOE 140 [176,177]. The mechanism for this ‘crosstalk’ has not yet been elucidated.

A further example of PAR_4_ crosstalk with the bradykinin B2 receptor was observed by Russell and colleagues in a rat knee model of joint inflammation [102]. In this model, it was found that (1) PAR_4_ activation by its peptide agonist, AYPGKF-NH_2_, induced sensitization of joint primary afferent sensory nerves in response to mechanical manipulation and that (2) the sensitization could be abrogated by HOE 140. Thus, the data indicate that the PAR_4_-mediated effect on the mechanosensitivity of knee joint afferent fibers is associated with bradykinin B2 receptor activation, pointing to a PAR_4_-B2 receptor crosstalk mechanism. Very likely, this kind of crosstalk between the bradykinin B2 receptor and PAR_2_ will be found in other situations. Whether the crosstalk involves a direct interaction between PAR_4_ and the bradykinin B2 receptor remains to be determined.

##### PAR_1_-inter-relationships with the serotonin 5HT2 receptor and the angiotensin AT1 receptor: impact on PAR_1_ transcription

Following disruption of the endothelium, sub-endothelial cell layers are exposed. This exposure promotes the activation of platelets and the initiation of the coagulation cascade resulting in the formation of thrombin and other members of the clotting enzyme family. Thrombin is present in balloon-injured vessels several weeks after injury [178] and, as a potent mitogen in fibroblasts and vascular smooth muscle cells (VSMCs) [179,180], thrombin has been implicated in the development of atherosclerotic lesions and restenosis by activation of its receptor, PAR_1_.

In normal arteries, PAR_1_ expression is detected in platelets, leukocytes, and endothelial cells, but it is low in VSMCs [23]. Notwithstanding, PAR_1_ activation in vessels causes an endothelium-independent contractile response, indicating that the low abundance smooth muscle PAR_1_ receptors are indeed functional. However, after vascular injury such as balloon angioplasty, PAR_1_ transcription is up-regulated in VSMCs [160,181], and this phenomenon is thought to be a key event in the development of vascular lesions and intimal thickening in response to thrombin [182]. The enhanced receptor expression is regulated by factors produced by the vascular wall and by activated platelets in the vicinity of the lesion. Besides basic fibroblast growth factor (bFGF) and platelet derived growth factor (PDGF)-AA [183], the GPCR agonists serotonin [184] and angiotensin II (AII) [185,186] have been shown to increase the expression of PAR_1_ mRNA in VSMCs. While the effect of serotonin (5HT) is mediated by the 5HT2 receptor and includes a pathway sensitive to tyrosine kinase inhibitors genistein and erbstatin A as well as inhibitors of PKC [184], AII increases PAR_1_ mRNA expression via the AT-1 receptor by a signalling route negatively regulated by PKC [185]. In addition, AII significantly increases (1) the thrombin-induced release of 6-keto-prostaglandin-1, and (2) the thrombin-induced contraction of endothelium-denuded aortic rings [186]. Thus, the upregulation of PAR_1_ expression by angiotensin II (AII) and 5HT at sites of vascular injury may potentiate the mitogenic and vasoconstrictor actions of thrombin in the vascular wall. This kind of PAR-GPCR inter-relationship does not require a physical interaction between the receptors.

##### PAR interactions with toll-like receptors

Toll-like receptors (TLRs) are pattern-recognition receptors (PRRs) that detect microbial structures (so-called, pathogen-associated molecular patterns, or ‘PAMPs’) and in turn activate cells of the ‘innate immune system’. The PAMPs are usually thought of as structural motifs shared between microbes [e.g. lipopolysaccharides (LPS) and lipopeptides]. However, as pointed out by Vogel and colleagues, by responding to pathogen- or tissue damage-derived proteinases, the PARs can be considered to represent ‘non-classical’ ‘Pattern-recognition receptors’ that also trigger the innate immune system [187-189]. PAR_2_, which is the best studied PAR with respect to an inflammatory response to microbial exposure, like the TLRs, is expressed highly in the respiratory and gastrointestinal tracts on epithelial cells, endothelial cells, macrophages, and dendritic cells. TLRs and PARs are distributed ubiquitously in the body and both PAR_2_ and the TLRs share the job of responding to pathogens. It was noted by Vogel and coworkers, that the inflammatory response caused by *Citrobacter rodentium* in mice is dependent both on TLRs and on PAR_2_[187]. Based on that association, it was proposed that there is signalling crosstalk between PAR_2_ and TLR4 [187-189]. Indeed, PAR_2_ activation has been shown to deliver intracellular signals that crosstalk with TLR signalling pathways [187-189] at least in part via a direct PAR_2_-TLR4 interaction [187]. Specifically, PAR_2_ activation and lipopolysaccharide (LPS) activation of TLR4 synergistically enhance inflammatory signalling in airway epithelial cells by raising the level of PAR expression and secretion of interleukin (IL)-8. The PAR_2_ activating peptide, SLIGKV-NH_2_, was capable of inducing NF-κB and NF-κB-dependent IL-1β mRNA expression was diminished in TLR4^−/−^ macrophages. In vivo, PAR_2_ activating peptide-induced footpad edema was significantly diminished in both TLR4^−/−^ and MyD88^−/−^ mice, supporting the concept of PAR_2_-TLR4 receptor cooperativity in which optimal PAR_2_ signalling leading to an inflammatory response requires TLR4 and MyD88. Zhou and colleagues [190] also reported a mutual regulation of TLR4-PAR_2_ expression in that LPS/TLR4 stimulation increases PAR_2_ expression on human colon cancer SW620 cells and a PAR_2_ agonist induces TLR4 mRNA. Moreover, the PAR_2_ activating peptide (SLIGKV-NH_2_) augmented LPS-induced IL-8 secretion and promoted proliferation and migration synergistically with TLR4 in SW620 cells [191]. Thus, there is crosstalk between PAR_2_ and TLR4 that involves both direct receptor interactions and indirect signal pathway crosstalk that result in an innate defense inflammatory response.

In addition to stimulating an inflammatory response, PAR_2_ activation is also known to cause ‘protective’ signaling in certain settings [67]. In this regard, the inflammatory cytokine response of primary murine peritoneal and bone marrow-derived macrophages to TLR4 was found to be diminished by PAR_2_ stimulation [190]. Treatment with LPS and the PAR_2_-activating peptides, SLIGKV-NH_2_ and 2-furoyl-LIGRLO-NH_2_, resulted in a concentration-dependent down-regulation of TNF-α, IL-6, and IL-12p40 mRNA, and an increase in IL-10. It was also observed that PAR_2_ activation of wild-type macrophages enhances LPS-induced expression of interleukins IL-4, IL-10, and IL-13, while suppressing expression of the proinflammatory cytokines TNF-α, IL-6, and IL-12. *In vitro* and *in vivo* PAR_2_ and TLR4 signalling pathways intersect such that PAR_2_ promotes development of an anti-inflammatory IL-10 response while dampening the helper T cell 1 (Th1)-like pro-inflammatory response induced by LPS. PAR_2_ activating peptides (SLIGKV-NH_2_ and 2-furoyl-LIGRLO-NH_2_) synergistically enhance LPS-induced mRNA expression of alternatively activated macrophage markers arginase-1, mannose receptor, and Ym-1 [189]. However, the mechanistic basis of these interactions remains to be elucidated.

Apart from TLR4, cooperative signalling convergence has also been observed between PAR_2_ and both TLR2 and TLR3 [188]. For mRNA induction of NF-κB-dependent IL-8, the cooperation between PAR_2_ and TLR3 (poly I:C activation) was highly synergistic. It was also found that PAR_2_-TLR3 coactivation can lead to differential signalling outcomes in TLR3-stimulated mucosal epithelial cells. Thus, although PAR_2_ and TLR3 synergize to up-regulate NF-κB-responsive genes, in the context of a response to viral infection in which TLR3 senses viral RNA, PAR_2_ stimulation of cultured lung A549 epithelial cells causes a reduced expression of TLR3-, and interferon-response-factor-3 (IRF-3)-driven genes, and a suppression of TLR3-inducible STAT1 activation [188]. Interestingly, these *in vitro* observations showing a negative impact of PAR_2_ activation on TLR3-induced gene expression in A549 and SW620 cells were supported by results obtained *in vivo*, demonstrating that PAR_2_^−/−^ mice were more susceptible to a pulmonary inflammatory response following intranasal infection with pseudomonas than wild type mice [191]. Thus, PAR_2_ activation can exert both positive and negative interactions when interacting with TLR signaling, depending on the identity of the TLR with which it interacts. It remains to be determined if the PAR_2_-TLR interactions observed when PAR_2_ is activated enzymatically will accurately reflect the observations that have been made with the use of the PAR_2_-activating peptides.

In addition to having an impact on bacterial and viral infection, interactions between TLRs and PARs also contribute to signal diversity in response to fungal infections caused by *Candida albicans* and *Aspergillus fumigates*[192]. These fungi activate PARs and trigger distinct signal transduction pathways involved in inflammation and immunity (1) by differentially regulating PAR expression through stimulating TLR2 and TLR4, both in polymorphonuclear neutrophils (PMNs) *in vitro* and in the stomach and lungs of infected mice, (2) by releasing PAR-regulating proteases from PMNs in a TLR-dependent manner and (3) by releasing fungal proteases that can cleave PARs and alter their capacity to signal. The signaling crosstalk between PARs 1 and 2 and the TLRs represents another instance of PAR-TLR interactions, but the precise mechanisms that lead to this signaling crosstalk in fungal infections have yet to be determined [192].

To sum up, PAR-TLR interactions, as hypothesized by Vogel and colleagues [187] have been documented both via direct (i.e. PAR-TLR interactions) and indirect (i.e. signal crosstalk) mechanisms in a number of settings ranging from the actions of lipopolysaccharide and other TLR-activating ligands in cell expression systems to the response of cells and tissues to TLR-activating ligands *in vivo*. These interactions can involve not only the cells of the innate immune response system (e.g. macrophages), but also tissue epithelial and vascular endothelial cells [193].

##### PAR interactions with NOD-like receptors

In addition to synergizing with the toll-like receptors, the PARs also appear able to interact with signalling via the NOD-like receptors (NLRs) which like the TLRs are also activated by pathogen-associated molecular patterns [194]. The mechanisms whereby the NLRs can synergize with PAR signalling, as observed for oral pathogens [195], remain to be determined.

#### PAR interactions with multi-subunit ion channel receptors and TRP ion channels

##### PARs 1 and 2 and the N-methyl-D-aspartate (NMDA) receptor

The effects of astrocytic PAR_1_ activation on neuronal health are complex and include both neuroprotective and neurotoxic activities [196-201]. This complicated situation is mainly due to the ability of PAR_1_ to trigger different signalling pathways in multiple cell types in the brain. At present, some of the PAR_1_-mediated neuronal effects are thought to depend on its ability to potentiate the function of the synaptic N-methyl-D-aspartate (NMDA) receptor [196,202]. The NMDA receptor is a ligand-gated ion channel that requires coactivation by two endogenous ligands, glutamate and either D-serine or glycine. The NMDA receptor plays a critical role in higher level brain processes and has been implicated for decades in neurological diseases such as stroke, traumatic brain injury, dementia and schizophrenia (for review see [203]). Specifically, several lines of evidence indicate that plasmin and thrombin can regulate the function of NMDA receptors through PAR_1_ activation. While tissue plasminogen activator (tPA)-activated plasmin has been suggested to induce PAR_1_-mediated regulation of NMDA receptor function in a manner relevant for synaptic plasticity and behaviour [204,205], NMDA receptor activity seems to be necessary for thrombin/PAR_1_-induced neurodegenerative effects under pathological conditions such as ischemia or hemorrhage [206,207]. For example, in granule cells of the dentate gyrus, a subset of neurons, Han et al. showed that PAR_1_ activation leads to cell depolarization and potentiation of synaptically activated NMDA receptor function [208]. This result supports the concept that PAR_1_ can enhance neuronal excitability, which may promote NMDA-receptor mediated neuronal damage [207]. Whether this enhancement is due to an effect of PAR_1_ on the NMDA receptor or via the ability of PAR_1_ to regulate neuronal TRPV channels (see below) remains to be determined.

There is growing evidence that astrocytes, a subset of glial cells, are capable of participating actively in neuronal function (for review see e.g.: [209]). In these cells PAR_1_ is able to trigger calcium signaling. Interestingly, Shigetomi et al. showed that under conditions when [Ca^2+^] is appropriately elevated, by activating PAR_1_, glutamate-NMDA receptor-mediated slow inward currents (SICs) in pyramidal neurons can be observed [210]. A further example for a PAR_1_-NMDA interplay in astrocytes was provided by Boven et al. who found that the NMDA receptor is involved in PAR_1_ mediated effects on gene expression including induction of inflammatory mediators, IL-1β and iNOS. This mechanism is thought to contribute to neuronal damage during human immunodeficiency virus (HIV)-encephalitis [211].

Of importance, PAR_2_, like PAR_1_, is widely expressed in the central nervous system under physiological conditions. PAR_2_ activation leads to a depolarization of hippocampal neurons and a paradoxical reduction in the occurrence of synaptically driven spontaneous action potentials. Gan et al. showed that PAR_2_ activation induces a profound long-term depression of synaptic transmission that is dependent on NMDA receptor activation and is sensitive to disruption of astrocytic function [212].

##### The P2X1 ion channel receptor and PAR_4_-α_2A_-adrenergic receptor crosstalk

Besides the crosstalk of PAR_1_ and PAR_4_ to regulate human platelet function and to affect signalling by other G protein-coupled receptors, complex interactions with ion channel receptors are also possible. This complexity is illustrated by the way PAR_4_ and α_2A_-adrenergic receptors can cooperate to cause aggregation of aspirin-treated human platelets [213]. This effect can reverse the otherwise beneficial therapeutic effects of aspirin, which irreversibly alkylates and inactivates human platelet cyclooxygenase. In such aspirin-treated platelets, cooperative signaling by PAR_4_ and the α_2A_-adrenergic receptor (but *not* PAR_1_) leads to the release of platelet dense-granule-stored adenosine triphosphate (ATP), which in turn triggers the P2X1 ATP-gated calcium ion channel to cause aggregation. It is this complex mechanism that can bypass the inhibitory effect of aspirin on platelet aggregation. This example is provided to indicate how complex and convoluted the interactions of PAR signalling can be and to alert the reader to the very rapid events that can accompany PAR signalling so as to affect multiple effector pathways simultaneously, even at the level of ion channel regulation.

##### PAR interaction with transient receptor potential (TRP) ion channels

Transient receptor potential (TRP) ion channels comprise a large 29-member family that regulate the transmembrane cellular influx of cations (mainly Na^+^; Ca^2+^) (for reviews, see: [214,215]). TRP channel activity can be modulated by receptor signaling triggered by both growth factor receptors and G protein-coupled receptors. In this regard, PARs are no exception, and their activation can influence TRP channel activity by a number of mechanisms involving: (1) stimulating the hydrolysis of phosphatidylinositol (4,5) bisphosphate (PIP2) to dissociate PIP2 from the channel, (2) release of the second messenger, diacylglycerol, that in turn can trigger PKC phosphorylation of the channel, (3) triggering tyrosine kinase-mediated channel phosphorylation and (4) generating inositol (1,4,5) trisphosphate (InsP3), the partner of diacylglycerol release that in turn elevates intracellular calcium to drive calmodulin-dependent changes in channel function. In principle, all of PARs 1, 2 and 4 could affect TRP channels by these mechanisms. The following paragraphs provide some examples, with a focus on PAR_2_-regulated TRPV1 and TRPV4 function.

At present, there is clear evidence that PAR_2_ is functionally involved in peripheral mechanisms of inflammation and pain [216-218], partly via sensitisation of the transient receptor potential vanilloid subfamily 1 (TRPV1) receptor [218-222]. TRPV1 (also designated capsaicin receptor or vanilloid receptor 1), a member of the TRPV group of transient receptor potential family of ion channels comprising 4 subtypes (TRPV1-TRPV4), functions as a sensor for thermal and acidic nociception and is known to be critically involved in the processing of somatic and visceral inflammatory pain [223,224]. Since inhibitors of phospholipase Cβ (PLCβ), protein kinase A (PKA), or PKC can abolish PAR_2_-mediated transient receptor potential sensitization *in vitro* and *in vivo*[219,220,225], it is evident that PAR_2_ induces receptor sensitization through a canonical PLC/Ca^2+^/PKC-signalling pathway. For instance, a trypsin-PAR_2_-TRPV1 axis has been shown to be linked to pain in pancreatitis [226,227]. In the skin, PAR_2_-triggered hypersensitivity to heat can be diminished by pharmacological inhibition of TRPV1 with capsazepine and is not observed in TRPV1 knockout mice [219,220]. In a relatively recently published report Suckow et al. provide evidence that PAR_2_-TRPV1 crosstalk mediates an extrinsic motor reflex pathway in the rat colon [228].

Activation of PAR_2_ is known to play a protective role in myocardial ischemia-reperfusion (I/R) injury [229-231]. In 2002, McLean et al. provided first evidence for an interaction between PAR_2_ and TRPV1 in this cardiovascular condition [232]. They showed that PAR_2_ activation causes endothelium-dependent coronary vasodilation that is preserved after I/R injury. Using hearts from TRPV1 knockout mice or wild-type mice it was found that PAR_2_-induced cardiac protection against I/R injury depends at least in part on PAR_2_ activation of TRPV1 via stimulation of the PKA or PKCϵ pathways leading to the sensitization of neuronal TRPV1 and a release of the inflammatory mediators, calcitonin gene-related peptide (CGRP) and substance P (SP) [233]. This PAR_2_-PKA/PKCϵ-TRPV1-CGRP/SP signalling route may serve as a promising pathway for the development of future multitarget therapies for cardiac injury and inflammation.

In a similar vein, Vellani et al. showed that PAR_1_ and PAR_4_, by activating PKCϵ, causes sensitization of TRPV1 and promotes the heat-dependent release of the pro-inflammatory neuropeptide CGRP in a sub-population of nociceptive neurons [234]. These data provide an explanation for the inflammatory effects of higher levels of thrombin and specific PAR_1/4_ agonists. Thus, following injury and rupture of blood vessels, the release of significant amounts of thrombin could act on nociceptive nerve terminals, sensitizing TRPV1 to heat stimuli and promoting the release of pro-inflammatory neuropeptides such as CGRP.

An instructive example of PAR-TRPV channel interactions has come from a study of the regulation of TRPV4 function by PAR_2_, which stimulates a sustained influx of calcium via the channel [235]. This sustained calcium influx results from a Src-mediated phosphorylation of a target tyrosine on TRPV4. Thus, tyrosine kinase phosphorylation of TRPV channels as well as protein kinase C-mediated regulation can lead to PAR-TRPV channel interactions. No doubt, other GPCRs can also cause comparable effects to regulate the TRPV channels.

In summary, activation of all of PARs 1, 2 and 4 can lead to a modulation of TRPV channel function, involving TRPV1, TRPV4 and even TRPA1 [236]. To date, this regulation has been found to result from the activation of kinase signalling pathways by the PARs that in turn target the TRPs, rather than via direct PAR-TRPV channel interactions. Whether the PARs can interact directly with TRP channels to regulate activity remains to be seen.

#### Crosstalk at the level of receptor trafficking

##### A role for PAR_2_ in membrane trafficking of PAR_4_

As already outlined above, overexpressed PAR_2_ and PAR_4_ are able to form heterodimer complexes in keratinocyte NCT-2544 cells and in human embryonal kidney HEK 293 T cells [166]. The data from this study provide evidence for a functional PAR-PAR interaction where PAR_2_-PAR_4_ heterodimer formation is facilitated by the plasma membrane delivery of PAR_4_ through disruption of its binding to the endoplasmic reticulum protein, COP1 β-subunit (β-COP1), and by the interactions of PAR_4_ with the chaperone protein 14-3-3ζ. Of note, the association of PAR_2_ with PAR_4_ markedly enhances PAR_4_-mediated ^3^H-InsP3 accumulation in NCT-2544 cells [166].

##### PAR association with cargo receptors (p23, p24A)

The underlying mechanistic basis for the internalization, recycling and lysosomal sorting of PARs is just beginning to emerge (for review see e.g.: [237]). P23 and p24A are transmembrane proteins [238] that function as coat protein receptors as well as cargo receptors by cycling between the endoplasmic reticulum (ER) and the Golgi apparatus. These proteins are involved in protein transport and quality control in the early secretory pathway [239]. Recently, Reiser and colleagues demonstrated that p23 and p24A interact with PAR_1_ and PAR_2_ and function as cargo receptors in the post-Golgi trafficking of PAR_1_ and PAR_2_[240,241]. Since intracellular trafficking of GPCRs regulates spatial and temporal receptor signalling, this crosstalk may be important for the physiological and pathophysiological functions of PAR_1_ and PAR_2_, respectively.

## Proteinase-activated receptor signalling, receptor dimerization and crosstalk: challenges for therapeutic drug development

### Receptor homo- and hetero-dimerization, PAR function and PARs as therapeutic targets

As outlined in previous sections, PARs are able to function both as monomeric receptors and as partners in a variety of PAR-PAR, PAR-GPCR and PAR-non-GPCR effector complexes. A key issue to deal with is the therapeutic relevance of this ability of PARs to form multimeric signalling complexes. This ‘pairing’ of G protein-coupled receptors has a substantial impact on the action of both PAR agonists and antagonists because of the ‘biased signalling’ that can ensue for either homo- or heterodimers [41]. This issue is relevant not only to the PARs themselves, but also to the potential ‘partners’ with which PARs may signal, since the non-PAR targets will also have their own set of agonists and antagonists.

This dimerization process is of importance for receptor maturation, internalization and downstream G protein coupling, as summarized in depth elsewhere [242-246]. The therapeutic relevance of this ‘dimerization’ process is that hetero-oligomers can have functional pharmacological characteristics that differ from the homo-oligomers, so as to cause distinct signalling and thus to alter a therapeutic impact [246]. The impact on signalling can be due to two issues: (1) the ability of only one of the two dimerized receptor subunits to generate a signal and (2) the triggering of unique ‘biased signalling’ [41,247] by individual agonists or antagonists that regulate one or both members of a receptor dimer.

### Therapeutic implications of PAR homo- and heterodimerization and ‘biased signalling’

The ability of selected ligands to drive different receptor conformations, so as to trigger distinct interactions with downstream effectors has been termed ‘functional selectivity’ or ‘biased signalling’ , as discussed above. In the case of receptor homo- or heterodimers, this flexibility is even more complex than in the case of a receptor signalling as a ‘monomer’ [247]. In the case of documented signalling by GPCR heterodimers (e.g. by angiotensin AT1 receptor-beta-2-adrenoceptors (ADRB2) dimers [248]), it has been possible to show that a validated antagonist for one receptor can affect the actions of the ‘partner’ in the dimer. For instance, the non-selective beta-adrenoceptor blocker, propranolol, is able to affect the ability of both angiotensin II and isoproterenol to attenuate agonist-stimulated myocyte contractility that is activated by angiotensin AT1-ADRB2 dimers [248]. Conversely, the angiotensin AT1 antagonist Valsartan can inhibit coupling of the beta-adrenoceptor to G_s_. These data, consistent with signalling by a heterodimeric receptor (AT1/ADRB2), depend on the well-established receptor antagonists, propranolol and valsartan. Unfortunately, this strategy is seriously hampered for evaluating the function of PAR homo- or heterodimers, because only a handful of therapeutically useful PAR antagonists are currently available [7], and because their mechanisms of PAR antagonism have been largely unexplored, except for their ability to block platelet aggregation (PAR_1_ antagonists) and calcium signalling via presumed G_q_ activation (PAR_1_ and PAR_2_ antagonists). The very recent elucidation of the structure of PAR_1_ bound to its antagonist Vorapaxar [249] will provide a scaffold to understand these mechanisms better and to facilitate the development of novel and specific PAR inhibitors. In addition, the crystallographic data seem to be helpful in identifying potential loops that will confer PAR receptor crosstalk via physical protein-protein interactions. For PAR_2_, in contrast with PAR_1_, the antagonists that have been developed so far have not yet proved of clinical utility in humans, although successful in diminishing inflammatory responses in rodent models of inflammation *in vivo*[250-253]. The promising PAR_2_ antagonists, Pepducin-P2pal-18S [250] and GB88 [251] have been found to be ‘biased antagonists’ that block G_q_-mediated calcium signalling. However, these antagonists do not affect either the agonist-stimulated interaction of PAR_2_ with beta-arrestin or the ability of PAR_2_ agonists to trigger receptor internalization [254]. Further, GB88 proves to be a ‘biased agonist’ that triggers both MAPK activation and an interaction of PAR_2_ with beta-arrestin [254,255]. What has not yet been evaluated in depth is the ability many of the available PAR_1_ antagonists to act as either full or biased antagonists for either PAR_1_ or PAR_2_ activation. More importantly, the effects of these compounds on signalling by PAR_1_/PAR_2_ heterodimers that can generate thrombin-stimulated signalling responses that are distinct from signalling activated by receptor homo-dimers [152] have not been considered. Thus, although of great therapeutic importance, the potential for the available PAR antagonists to affect signalling crosstalk between either (1) the PARs themselves (e.g. as independent crosstalk pathways or as heterodimers) or (2) via transactivation of other receptors, like the one for EGF (below), remains to be fully explored.

### PARs as therapeutic targets

The knowledge of the ability of PARs to cross-activate other receptor kinases or to synergise and/or form heterodimers with other members of the PAR family in driving specific pathophysiologic processes/diseases may possibly be exploited for a therapeutic benefit. In the last part of this review we would like to highlight those interactions that may have some pathophysiologic relevance and speculate as to whether one can target these signalling crosstalk interactions pharmacologically to benefit patients. In cases where both signalling partners have been identified, a combined drug approach for multi-target therapy seems promising. As already outlined above, to date no PAR-targeting drug has yet found its way into routine use in the clinic, although a number of PAR_1_ antagonists have been evaluated. Therefore, the following sections deal in a speculative way with the various settings in which targeting the PARs may prove of therapeutic value. It is to be emphasized that the considerations outlined in the previous paragraphs will bear directly on the development of PAR-targeted therapeutic agents for the clinical situations to be described.

#### Cardiovascular disease

A disease state, for which PAR-targeted intervention seems likely is cardiac injury where PAR_1_ and PAR_4_ signalling via EGFR transactivation contributes to the regulation of cardiac physiological and pathophysiological functions and remodeling, while activation of PAR_2_ plays a protective role in myocardial ischaemia-reperfusion injury [229-231] through an interaction between PAR_2_ and TRPV1 [232,233]. In addition, the PAR_2_-PKA/PKCϵ-TRPV1-CGRP/SP pathway may serve as a promising target for the development of future multitarget therapies for cardiac injury and inflammation [232,233].

In the regulation of vascular responses/atherosclerosis/stenosis, the combined use of specific PAR antagonists may be useful to block the functional coupling between PAR_1_ and PAR_2_ on vascular smooth muscle cells. This coupling appears to be responsible for the ability of PAR_2_ to regulate the PAR_1_ hyperplastic response to arterial injury leading to stenosis [150]. The prevention of stenosis may also be achievable with the combined use of PAR_1_ antagonists and the ATII receptor antagonists, Losartan, Valsartan or Irbesartan, or serotonin receptor blockers like the second-generation serotonin 5HT3 receptor antagonist Palonosetron. The idea behind this suggestion is to prevent upregulation of PAR_1_ expression/activity by AII and 5HT at sites of vascular injury. This upregulation may potentiate the mitogenic and constrictor actions of thrombin.

#### Lung fibrosis

PAR activation with or without transactivation of EGFR is also of importance in the pathophysiology of the lung, particularly in lung fibrosis. The use of PAR_4_-, PAR_2_-, and/or PAR_1_-specific antagonists along with EGFR blockers may be considered here since (1) PAR_4_-mediated EGFR signalling promotes alveolar epithelial-mesenchymal transition, an important mechanism in pulmonary lung fibrosis [108], (2) PAR_2_-mediated EGFR activation promotes the release of profibrotic IL-8 in human lung epithelial cells [109], and (3) PAR_1_-mediated enhancement of αVβ6-dependent TGF-β activation results in activation of the ALK5 kinase [50] and consequently profibrotic TGF-β responses. Since an overstimulation of PAR_1_-mediated enhancement of αVβ6-dependent TGF-β activation promotes acute lung injury [61], a combined therapy with PAR_1_ antagonists and TGF-β signalling inhibitors (see below) may be considered.

PAR_1_ and EGFR activation have been shown to stimulate migration of cultured HaCaT keratinocytes [96]. Hence, in patients that suffer from wound healing problems, e.g. diabetic patients, combined treatment with PAR_1_ + EGFR agonists could be envisaged. If overstimulation of this process is involved in scarring then treatment with PAR_1_ + EGFR antagonists could be considered as an option.

#### Arthritis

Work with PAR_2_-null mice has identified PAR_2_ as a potential therapeutic target for arthritis [103]. PAR_1_ is also thought to be involved. Since a role for PAR_1_-EGFR interplay in rheumatoid arthritis has been suggested [97] it may be worthwhile to test in a clinical study a combination of PAR_1_ and EGFR antagonists as outlined above.

#### Cancer

As mentioned in previous sections, EGFR transactivation in response to the stimulation of PARs occurs in a large number of cancers and is believed to contribute to cancer development and progression. Hence, the therapeutic targeting of PAR-EGFR interactions appears to be an extremely promising strategy. With respect to the progression of colon cancer, this mechanism includes PAR_2_-mediated EGFR transactivation and a subsequent increase of COX-2 expression in colonic epithelial cancer cells [127]. Moreover, in colon cancer cells, PAR_1_ and PAR_2_ induce migratory and proliferative effects that involve transactivation of the EGFR and activation of p42/p44 MAPK signalling pathways [112-114]. Likewise, activation of PAR_1_ by thrombin induces persistent EGFR and ErbB-2 transactivation, sustained p42/p44 MAPK signalling, and invasion in breast cancer cells [122]. The EGFR transactivation by PAR_1_ or PAR_2_ leading to COX-2 expression [124], enhanced cell proliferation in colon carcinoma cells [112,113] and cell migration in renal carcinoma cells [111] is dependent on matrix metalloproteinase (MMP) activity. Hence, pharmacologic targeting with PAR_1_/PAR_2_ and EGFR antagonists may be supplemented with MMP- and possibly COX-2 inhibitors.

In liver carcinoma cells, PAR_2_ triggers transactivation of the tyrosine kinase receptor, Met, to promote cell migration and invasion [54,55] and exhibits signalling crosstalk with the PDGFR to induce phosphorylation and activation of the PDGFR [53]. This result suggests that the combined use of a PAR_2_ antagonist together with inhibitors for Met or the PDGFR will prove of value in the anti-metastatic therapy of hepatocellular carcinoma. The combined use of PAR_1_ and PAR_2_ antagonists my be beneficial in malignant melanoma since studies on melanoma cells have indicated that stimulation of cell motility by thrombin requires not only the activation of PAR_1_ but also the simultaneous activation of PAR_2_[137]. As mentioned above, our own observations indicate that PAR_2_ expression is required for full-blown TGF-β/ALK5-induced migratory responses *in vitro.* Preclinical studies have provided convincing evidence that targeting the TGF-β pathway is able to inhibit tumor growth and metastasis *in vivo*[256]. For instance, small molecule inhibitors that target the kinase activity of TGF-βRI/ALK5 have been evaluated in preclinical mouse models of cancer (SD-208, SX-007, LY2109761) or are already being tested in clinical studies in cancer patients [(LY573636, LY2157299), ([256] and references therein)]. One hopes that a therapy combining PAR_2_ antagonists with (small molecule) kinase inhibitors may exhibit synergistic effects in treating metastatic disease of late-stage carcinomas.

In the intestine, PAR_2_ participates in the progression from chronic inflammation to colon cancer by crosstalk with PGE2/EP signalling [127]. Disrupting this crosstalk with PAR_2_ and/or PGE/EP inhibitors could potentially represent a powerful approach in preventing colon carcinoma at a very early step.

A PAR_1_/PAR_4_ two-receptor system has been demonstrated to mediate a closely related thrombin-induced signalling and cell migration process in both astrocytoma [138] and hepatocellular carcinoma [158]. If this response can be shown to contribute to tumor progression/metastasis, it may be worth targeting the cancer with agents that disrupt the PAR_1_-PAR_4_ interactions. In summary, in the oncology field a number of possible settings can be envisaged for the use of agents that target PAR-stimulated receptor transactivation processes.

#### Inflammation, pain and infection

PAR_1_, PAR_2_, and PAR_4_ have all been implicated in inflammation and infection, mostly with pro-inflammatory roles. The PAR_1_ and PAR_4_-mediated sensitization of TRPV1 resulting in the heat-dependent release of the pro-inflammatory neuropeptide CGRP in a sub-population of nociceptive neurons [234], may be targeted with PAR_1_/PAR_4_ and TRPV1 inhibitors, while the synergistic PAR_2_/LPS enhancement of inflammatory signalling in airway epithelial cells [188] may be blocked by combining a PAR_2_ and a TLR4 inhibitor.

Pro-inflammatory effects of PAR_4_ activation have been reported to be dependent on an interaction of PAR_4_ with the bradykinin B2 receptor. Administration of peptide-type B2 receptor antagonists (HOE-140 [176,177], NPC 567, or CP-0127) together with a PAR_4_ receptor antagonist may be a promising approach to the treatment of joint inflammation and joint primary afferent activity in response to mechanical stimuli. In a sepsis mouse model, PAR_1_ agonists can have a protective effect on endothelial barrier function and survival in mice [149]. In that study, the PAR_1_ agonist promoted transactivation of PAR_2_ by PAR_1_ and this transactivation switched the physiological response of the endothelial cells from barrier disruptive to barrier protective. That kind of response reversal for PAR_1_ signalling can also depend on the setting of PAR_1_ activation (caveolar vs not) and on the activating enzyme (e.g. direct PAR_1_ activation by thrombin promotes endothelial barrier disruption, whereas receptor activation by activated protein-C results in increased barrier function) [15,18]. A barrier-protective outcome may also result from the inhibition of the interaction of PAR_1_ with PAR_3_, the latter of which alters the selectivity of PAR_1_ for G_13_ coupling and promotes endothelial barrier dysfunction.

Activation of PAR_2_ has been implicated in pain in arthritis [100,101,103], pancreatitis [226,227], PAR_2_-triggered hypersensitivity to heat in the skin, and in neuropathic pain induced by paclitaxel [236]. Since in some cases the pain could be diminished by pharmacologic inhibition of TRPV1 with capsazepine, a more effective suppression of pain may be achieved with specific PAR_2_ antagonists once these have successfully passed clinical trials.

#### Neurodegeneration

NMDA receptor activity seems to be necessary for thrombin/PAR_1_-induced neurodegenerative effects under certain pathological conditions [206,207]. Specific PAR_1_ antagonists, in addition to NMDA receptor blockers in clinical use such as Ketamine would very likely be useful in preventing PAR_1_ from inducing NMDA receptor-mediated neuronal damage resulting from ischemia or haemorrhage.

## Summing up

This review provides a broad overview of what is known about the impact of PAR receptor-receptor interactions, either via direct or indirect mechanisms, on the regulation of cell and tissue function. From the variety of these receptor interactions and their diverse physiological and pathological roles it becomes clear that targeting this PAR-receptor crosstalk represents a promising but so far neglected strategy for modulating PAR signalling in disease. As outlined in a recent review on a related topic, a better understanding of the mechanism(s) of transactivation will provide novel possibilities for blocking the actions of PAR agonists and, as a consequence, their pathophysiological role in a range of diseases [257]. Depending on the nature of the interactions it may suffice to target only the PAR to prevent subsequent transactivation of the partner receptor. Alternatively, it may be necessary to block both the PAR and its interaction partner(s), or its/their downstream pathway(s), simultaneously in order to enhance the therapeutic efficacy. Although most of the above therapeutic considerations still remain speculative, evaluating them in (pre)clinical studies would add another dimension to PAR-directed drug therapy.

## Abbreviations

A: Alanine; R: Arginine; O: Ornithine; AC: Amino acids; 5HT: Serotonin; AII: Angiotensin II; ADP: Adenosine diphosphate; ADRB2: Beta-2-adrenoceptor; ATP: Adenosine- 5′-triphosphate; ALK5: Activin-like kinase 5 (also known as transforming growth factor beta receptor I; TGF-βRI); APC: Activated protein C; AT1: Angiotensin receptor subtype 1; B2 receptor: Bradykinin B2 receptor; BMP: Bone morphogenetic protein; BRET: Bioluminescence resonance energy transfer; CGRP: Calcitonine gene related-peptide; CCA: Cholangiocarcinoma; CCL2: Chemokine (C-C motif) ligand 2 [also referred to as monocyte chemotactic protein-1 (MCP-1)]; COX: Cyclooxygenase; ECs: Endothelial cells; EGFR/ErbB-1: Epidermal growth factor receptor; a member of the erythroblastosis homologue B (ErbB) family of receptor tyrosine kinases; El: Extracellular loop; EMT: Epithelial-mesenchymal transition; EP: Prostanoid receptor (prostaglandin receptor); ErbB-2: A second member of the ErbB family of receptor tyrosine kinases, also designated human epidermal growth factor receptor 2 (HER2); ER: Endoplasmic reticulum; ET-1: Endothelin-1; FGFR: Fibroblast growth factor receptor; FRET: Fluorescence resonance energy transfer; GPCR: G protein-coupled receptor; G protein: Heterotrimeric guanyl nucleotide-binding protein; HCC: Hepatocellular carcinoma; HIV: Human immunodeficiency virus; HUVECs: Human umbilical vein endothelial cells; ICAM-1: Intracellular adhesion molecule 1 (also known as CD 54, cluster of differentiation 54); IGFR: Insulin-like growth factor receptor; IL: Interleukin; Il: Intracellular loop; InsP3: Inositol (1,4,5) trisphosphate; I/R: Ischemia-reperfusion; IRF-3: Interferon-response-factor-3; KLK: Kallikrein-related peptidase family that includes prostate-specific antigen (PSA); LPA: Lysophosphatitic acid; LPS: Lipopolysaccharide; MAPK: Mitogen-activated protein kinase; Met: Hepatocyte growth factor (HGF) receptor; MKP-1: Mitogen-activated protein kinase phosphatase 1; MMP1: Matrix metalloproteinase-1; NF-κB: Nuclear factor 'kappa-light-chain-enhancer' of activated B-cells; NMDA: N-methyl-D-aspartate; NLR: NOD-like receptor, nucleotide oligomerization domain receptor; P2X1 receptor: ATP-gated cation channel; P2Y12: Purinergic receptor and chemoreceptor for ADP; PAECs: Pulmonary artery endothelial cells; PAMP: Pathogen-associated molecular pattern; PAR: Proteinase-activated receptor; PAR-APs: PAR-activating peptides; PDGFR: Platelet derived growth factor receptor; PGE2: Prostaglandin E-2; PI3K: Phosphatidylinositol 3-kinase; PIP2: Phosphatidylinositol (4,5) bisphosphate; PK: Plasma kallikrein; PKA: Protein kinase A, also known as cAMP-dependent protein kinase; PKC: Protein kinase C; PLA2: Phospholipase A2; PLC: Phospholipase C; PMNs: Polymorphonuclear neutrophils; PRRs: Pattern-recognition receptors; PTPs: Protein tyrosine phosphatases; RCC: Renal cell carcinoma; Rho: GTPase that belongs to the Rho family of GTPases; ROCK: Rho-associated protein kinase belonging to the family of serine/threonine-specific protein kinases; ROS: Reactive oxygen species; RSTK: Receptor serine/threonine kinase; RTK: Receptor tyrosine kinase; SICs: Slow inward currents; SMADs: Intracellular proteins that transduce TGF-beta receptor signals to trigger nuclear transcription; Src: Non receptor tyrosine kinase; SP: Substance P; TF: Tissue factor; TGF: Transforming growth factor; TK: Tissue kallikrein (now known as Kallikrein-related peptidases or KLK); TL: Tethered ligand; TLR: Toll like receptor; TM: Seven transmembrane helix; tPA: Tissue plasmin activator; TRPA: Transient receptor potential ankyrin A; TRPV: Transient receptor potential vanilloid; VEGFR: Vascular endothelial growth factor receptor; VSMCs: Vascular smooth muscle cells.

## Competing interests

The authors declare that they have no competing interests.

## Authors’ contributions

FG, HU, US, MDH and RK drafted and wrote the manuscript. All authors read and approved the final manuscript.

## References

[B1] Alexander SP Mathie A Peters JA Guide to receptors and channels (GRAC), 3rd edition Br J Pharmacol 2008 153 Suppl 2 S1 S209 18347570 10.1038/sj.bjp.0707746 PMC2275471

[B2] Hollenberg M Compton S International union of pharmacology. XXVIII. Proteinase-activated receptors Pharmacol Rev 2002 54 203 217 10.1124/pr.54.2.203 12037136

[B3] Ossovskaya V Bunnett N Protease-activated receptors: contribution to physiology and disease Physiol Rev 2004 84 579 621 10.1152/physrev.00028.2003 15044683

[B4] Ramachandran R Hollenberg M Proteinases and signalling: pathophysiological and therapeutic implications via PARs and more Br J Pharmacol 2008 153 Suppl 1 S263 S282 18059329 10.1038/sj.bjp.0707507 PMC2268078

[B5] Steinhoff M Buddenkotte J Shpacovitch V Rattenholl A Moormann C Vergnolle N Luger T Hollenberg M Proteinase-activated receptors: transducers of proteinase-mediated signaling in inflammation and immune response Endocr Rev 2005 26 1 43 15689571 10.1210/er.2003-0025

[B6] Adams MN Ramachandran R Yau MK Suen JY Fairlie DP Hollenberg MD Hooper JD Structure, function and pathophysiology of protease activated receptors Pharmacol Ther 2011 130 248 282 10.1016/j.pharmthera.2011.01.003 21277892

[B7] Ramachandran R Noorbakhsh F Defea K Hollenberg MD Targeting proteinase-activated receptors: therapeutic potential and challenges Nat Rev Drug Discov 2012 11 69 86 10.1038/nrd3615 22212680

[B8] Bockaert J Pin JP Molecular tinkering of G protein-coupled receptors: an evolutionary success EMBO J 1999 18 1723 1729 10.1093/emboj/18.7.1723 10202136 PMC1171258

[B9] Wettschureck N Offermanns S Mammalian G proteins and their cell type specific functions Physiol Rev 2005 85 1159 1204 10.1152/physrev.00003.2005 16183910

[B10] Vu T Hung D Wheaton V Coughlin S Molecular cloning of a functional thrombin receptor reveals a novel proteolytic mechanism of receptor activation Cell 1991 64 1057 1068 10.1016/0092-8674(91)90261-V 1672265

[B11] Mihara K Ramachandran R Renaux B Saifeddine M Hollenberg MD Neutrophil elastase and proteinase-3 trigger G-protein biased signaling through proteinase activated receptor-1 (PAR1) J Biol Chem 2013 Sep 19. [Epub ahead of print] 10.1074/jbc.M113.483123 PMC3829148 24052258

[B12] Boire A Covic L Agarwal A Jacques S Sherifi S Kuliopulos A PAR1 is a matrix metalloprotease-1 receptor that promotes invasion and tumorigenesis of breast cancer cells Cell 2005 120 303 313 10.1016/j.cell.2004.12.018 15707890

[B13] Trivedi V Boire A Tchernychev B Kaneider NC Leger AJ O'Callaghan K Covic L Kuliopulos A Platelet matrix metalloprotease-1 mediates thrombogenesis by activating PAR1 at a cryptic ligand site Cell 2009 137 332 343 10.1016/j.cell.2009.02.018 19379698 PMC2807741

[B14] Ramachandran R Mihara K Chung H Renaux B Lau CS Muruve DA DeFea KA Bouvier M Hollenberg MD Neutrophil elastase acts as a biased agonist for proteinase-activated receptor-2 (PAR2) J Biol Chem 2011 286 24638 24648 10.1074/jbc.M110.201988 21576245 PMC3137039

[B15] Schuepbach RA Madon J Ender M Galli P Riewald M Protease-activated receptor-1 cleaved at R46 mediates cytoprotective effects J Thromb Haemost 2012 10 1675 1684 10.1111/j.1538-7836.2012.04825.x 22712885 PMC3419798

[B16] Nakanishi-Matsui M Zheng YW Sulciner DJ Weiss EJ Ludeman MJ Coughlin SR PAR3 is a cofactor for PAR4 activation by thrombin Nature 2000 404 609 613 10.1038/35007085 10766244

[B17] Ostrowska E Reiser G The protease-activated receptor-3 (PAR-3) can signal autonomously to induce interleukin-8 release Cell Mol Life Sci 2008 65 970 981 10.1007/s00018-008-7555-y 18264801 PMC11131819

[B18] Mosnier LO Sinha RK Burnier L Bouwens EA Griffin JH Biased agonism of protease-activated receptor 1 by activated protein C caused by noncanonical cleavage at Arg46 Blood 2012 120 5237 5246 10.1182/blood-2012-08-452169 23149848 PMC3537315

[B19] Rasmussen UB Vouret-Craviari V Jallat S Schlesinger Y Pagès G Pavirani A Lecocq JP Pouysségur J Van Obberghen-Schilling E cDNA cloning and expression of a hamster alpha-thrombin receptor coupled to Ca2+ mobilization FEBS Lett 1991 288 123 128 10.1016/0014-5793(91)81017-3 1652467

[B20] Scarborough RM Naughton MA Teng W Hung DT Rose J Vu TK Wheaton VI Turck CW Coughlin SR Tethered ligand agonist peptides. Structural requirements for thrombin receptor activation reveal mechanism of proteolytic unmasking of agonist function J Biol Chem 1992 267 13146 13149 1320011

[B21] Hansen KK Saifeddine M Hollenberg MD Tethered ligand-derived peptides of proteinase-activated receptor 3 (PAR3) activate PAR1 and PAR2 in Jurkat T cells Immunology 2004 112 183 190 10.1111/j.1365-2567.2004.01870.x 15147561 PMC1782474

[B22] Kaufmann R Schulze B Krause G Mayr LM Settmacher U Henklein P Proteinase-activated receptors (PARs)–the PAR3 Neo-N-terminal peptide TFRGAP interacts with PAR1 Regul Pept 2005 125 61 66 10.1016/j.regpep.2004.07.032 15582715

[B23] Macfarlane SR Seatter MJ Kanke T Hunter GD Plevin R Proteinase-activated receptors Pharmacol Rev 2001 53 245 282 11356985

[B24] Russo A Soh UJ Trejo J Proteases display biased agonism at protease-activated receptors: location matters! Mol Interv 2009 9 87 96 10.1124/mi.9.2.8 19401541 PMC3139377

[B25] Coughlin SR Protease-activated receptors in hemostasis, thrombosis and vascular biology J Thromb Haemost 2005 3 1800 1814 10.1111/j.1538-7836.2005.01377.x 16102047

[B26] Hung DT Wong YH Vu TK Coughlin SR The cloned platelet thrombin receptor couples to at least two distinct effectors to stimulate phosphoinositide hydrolysis and inhibit adenylyl cyclase J Biol Chem 1992 267 20831 20834 1328213

[B27] Rahman A True AL Anwar KN Ye RD Voyno-Yasenetskaya TA Malik AB Galpha(q) and Gbetagamma regulate PAR-1 signaling of thrombin-induced NF-kappaB activation and ICAM-1 transcription in endothelial cells Circ Res 2002 91 398 405 10.1161/01.RES.0000033520.95242.A2 12215488

[B28] Ramachandran R Mihara K Mathur M Rochdi MD Bouvier M Defea K Hollenberg MD Agonist-biased signaling via proteinase activated receptor-2: differential activation of calcium and mitogen-activated protein kinase pathways Mol Pharmacol 2009 76 791 801 10.1124/mol.109.055509 19605524 PMC2769049

[B29] Lefkowitz RJ Shenoy SK Transduction of receptor signals by beta-arrestins Science 2005 308 512 517 10.1126/science.1109237 15845844

[B30] Shenoy SK Lefkowitz RJ Seven-transmembrane receptor signaling through beta-arrestin Sci STKE 2005 2005 cm10 16267056 10.1126/stke.2005/308/cm10

[B31] Defea K Beta-arrestins and heterotrimeric G-proteins: collaborators and competitors in signal transduction Br J Pharmacol 2008 153 Suppl 1 S298 S309 18037927 10.1038/sj.bjp.0707508 PMC2268080

[B32] Chen CH Paing MM Trejo J Termination of protease-activated receptor-1 signaling by beta-arrestins is independent of receptor phosphorylation J Biol Chem 2004 279 10020 10031 14699102 10.1074/jbc.M310590200

[B33] Ge L Shenoy SK Lefkowitz RJ DeFea K Constitutive protease-activated receptor-2-mediated migration of MDA MB-231 breast cancer cells requires both beta-arrestin-1 and −2 J Biol Chem 2004 279 55419 55424 10.1074/jbc.M410312200 15489220

[B34] Wang P Jiang Y Wang Y Shyy JY DeFea KA Beta-arrestin inhibits CAMKKbeta-dependent AMPK activation downstream of protease-activated-receptor-2 BMC Biochem 2010 11 36 10.1186/1471-2091-11-36 20858278 PMC2955585

[B35] Zoudilova M Kumar P Ge L Wang P Bokoch GM DeFea KA Beta-arrestin-dependent regulation of the cofilin pathway downstream of protease-activated receptor-2 J Biol Chem 2007 282 20634 20646 10.1074/jbc.M701391200 17500066

[B36] Weis WI Kobilka BK Structural insights into G-protein-coupled receptor activation Curr Opin Struct Biol 2008 18 734 740 10.1016/j.sbi.2008.09.010 18957321 PMC4019673

[B37] Gether U Kobilka BK G protein-coupled receptors. II. Mechanism of agonist activation J Biol Chem 1998 273 17979 17982 10.1074/jbc.273.29.17979 9660746

[B38] de Haën C The non-stoichiometric floating receptor model for hormone sensitive adenylyl cyclase J Theor Biol 1976 58 383 400 10.1016/S0022-5193(76)80126-9 181640

[B39] Jacobs S Cuatrecasas P The mobile receptor hypothesis and "cooperativity" of hormone binding. Application to insulin Biochim Biophys Acta 1976 433 482 495 10.1016/0005-2736(76)90275-3 179590

[B40] Kenakin T Miller LJ Seven transmembrane receptors as shapeshifting proteins: the impact of allosteric modulation and functional selectivity on new drug discovery Pharmacol Rev 2010 62 265 304 10.1124/pr.108.000992 20392808 PMC2879912

[B41] Kenakin TP Biased signalling and allosteric machines: new vistas and challenges for drug discovery Br J Pharmacol 2012 165 1659 1669 10.1111/j.1476-5381.2011.01749.x 22023017 PMC3372820

[B42] Little PJ Burch ML Al-aryahi S Zheng W The paradigm of G protein receptor transactivation: a mechanistic definition and novel example ScientificWorld Journal 2011 11 709 714 21442148 10.1100/tsw.2011.75 PMC5720110

[B43] Feistritzer C Riewald M Endothelial barrier protection by activated protein C through PAR1-dependent sphingosine 1-phosphate receptor-1 crossactivation Blood 2005 105 3178 3184 10.1182/blood-2004-10-3985 15626732

[B44] Finigan JH Dudek SM Singleton PA Chiang ET Jacobson JR Camp SM Ye SQ Garcia JG Activated protein C mediates novel lung endothelial barrier enhancement: role of sphingosine 1-phosphate receptor transactivation J Biol Chem 2005 280 17286 17293 10.1074/jbc.M412427200 15710622

[B45] Guo H Zhao Z Yang Q Wang M Bell RD Wang S Chow N Davis TP Griffin JH Goldman SA Zlokovic BV An activated protein C analog stimulates neuronal production by human neural progenitor cells via a PAR1-PAR3-S1PR1-Akt pathway J Neurosci 2013 33 6181 6190 10.1523/JNEUROSCI.4491-12.2013 23554499 PMC3707621

[B46] Daub H Weiss F Wallasch C Ullrich A Role of transactivation of the EGF receptor in signalling by G-protein-coupled receptors Nature 1996 379 557 560 10.1038/379557a0 8596637

[B47] Daub H Wallasch C Lankenau A Herrlich A Ullrich A Signal characteristics of G protein-transactivated EGF receptor EMBO J 1997 16 7032 7044 10.1093/emboj/16.23.7032 9384582 PMC1170306

[B48] Ferguson SS Barak LS Zhang J Caron MG G-protein-coupled receptor regulation: role of G-protein-coupled receptor kinases and arrestins Can J Physiol Pharmacol 1996 74 1095 1110 10.1139/y96-124 9022829

[B49] Wetzker R Böhmer F Transactivation joins multiple tracks to the ERK/MAPK cascade Nat Rev Mol Cell Biol 2003 4 651 657 10.1038/nrm1173 12923527

[B50] Burch ML Osman N Getachew R Al-Aryahi S Poronnik P Zheng W Hill MA Little PJ G protein coupled receptor transactivation: extending the paradigm to include serine/threonine kinase receptors Int J Biochem Cell Biol 2012 44 722 727 10.1016/j.biocel.2012.01.018 22326998

[B51] Chandrasekharan UM Waitkus M Kinney CM Walters-Stewart A DiCorleto PE Synergistic induction of mitogen-activated protein kinase phosphatase-1 by thrombin and epidermal growth factor requires vascular endothelial growth factor receptor-2 Arterioscler Thromb Vasc Biol 2010 30 1983 1989 10.1161/ATVBAHA.110.212399 20671228 PMC2956164

[B52] Siegbahn A Johnell M Nordin A Aberg M Velling T TF/FVIIa transactivate PDGFRbeta to regulate PDGF-BB-induced chemotaxis in different cell types: involvement of Src and PLC Arterioscler Thromb Vasc Biol 2008 28 135 141 17991872 10.1161/ATVBAHA.107.155754

[B53] Mußbach F Henklein P Settmacher U Böhmer F-D Kaufmann R RTKs, ROS and PTPs are involved in signal transduction of proteinase-activated receptors 1, 2 and 4 in HEP-3B hepatocellular carcinoma cells. 16th Joint Meeting of the Signal Transduction Society (STS) 2012 Weimar, Germany: Cell Communication & Signaling 110

[B54] Kaufmann R Oettel C Horn A Halbhuber K Eitner A Krieg R Katenkamp K Henklein P Westermann M Böhmer F Met receptor tyrosine kinase transactivation is involved in proteinase-activated receptor-2-mediated hepatocellular carcinoma cell invasion Carcinogenesis 2009 30 1487 1496 10.1093/carcin/bgp153 19546160

[B55] Kaufmann R Hascher A Mußbach F Henklein P Katenkamp K Westermann M Settmacher U Proteinase-activated receptor 2 (PAR(2)) in cholangiocarcinoma (CCA) cells: effects on signaling and cellular level Histochem Cell Biol 2012 138 913 924 10.1007/s00418-012-1006-4 22892662

[B56] Du J Sperling LS Marrero MB Phillips L Delafontaine P G-protein and tyrosine kinase receptor cross-talk in rat aortic smooth muscle cells: thrombin- and angiotensin II-induced tyrosine phosphorylation of insulin receptor substrate-1 and insulin-like growth factor 1 receptor Biochem Biophys Res Commun 1996 218 934 939 10.1006/bbrc.1996.0165 8579617

[B57] Delafontaine P Growth factors and vascular smooth muscle cell growth responses Eur Heart J 1998 19 Suppl G G18 G22 9717051

[B58] Delafontaine P Anwar A Lou H Ku L G-protein coupled and tyrosine kinase receptors: evidence that activation of the insulin-like growth factor I receptor is required for thrombin-induced mitogenesis of rat aortic smooth muscle cells J Clin Invest 1996 97 139 145 10.1172/JCI118381 8550825 PMC507072

[B59] Burch ML Ballinger ML Yang SN Getachew R Itman C Loveland K Osman N Little PJ Thrombin stimulation of proteoglycan synthesis in vascular smooth muscle is mediated by protease-activated receptor-1 transactivation of the transforming growth factor beta type I receptor J Biol Chem 2010 285 26798 26805 10.1074/jbc.M109.092767 20571025 PMC2930678

[B60] Derynck R Akhurst RJ Balmain A TGF-beta signaling in tumor suppression and cancer progression Nat Genet 2001 29 117 129 10.1038/ng1001-117 11586292

[B61] Jenkins RG Su X Su G Scotton CJ Camerer E Laurent GJ Davis GE Chambers RC Matthay MA Sheppard D Ligation of protease-activated receptor 1 enhances alpha(v)beta6 integrin-dependent TGF-beta activation and promotes acute lung injury J Clin Invest 2006 116 1606 1614 10.1172/JCI27183 16710477 PMC1462943

[B62] Nguyen QD De Wever O Bruyneel E Hendrix A Xie WZ Lombet A Leibl M Mareel M Gieseler F Bracke M Gespach C Commutators of PAR-1 signaling in cancer cell invasion reveal an essential role of the Rho-Rho kinase axis and tumor microenvironment Oncogene 2005 24 8240 8251 10.1038/sj.onc.1208990 16091733

[B63] Tsuchida K Nakatani M Hitachi K Uezumi A Sunada Y Ageta H Inokuchi K Activin signaling as an emerging target for therapeutic interventions Cell Commun Signal 2009 7 15 10.1186/1478-811X-7-15 19538713 PMC2713245

[B64] Liu Y Ren W Warburton R Toksoz D Fanburg BL Serotonin induces Rho/ROCK-dependent activation of Smads 1/5/8 in pulmonary artery smooth muscle cells FASEB J 2009 23 2299 2306 10.1096/fj.08-127910 19244313

[B65] Perillan PR Chen M Potts EA Simard JM Transforming growth factor-beta 1 regulates Kir2.3 inward rectifier K + channels via phospholipase C and protein kinase C-delta in reactive astrocytes from adult rat brain J Biol Chem 2002 277 1974 1980 10.1074/jbc.M107984200 11713246

[B66] Takizawa T Tamiya M Hara T Matsumoto J Saito N Kanke T Kawagoe J Hattori Y Abrogation of bronchial eosinophilic inflammation and attenuated eotaxin content in protease-activated receptor 2-deficient mice J Pharmacol Sci 2005 98 99 102 10.1254/jphs.SCZ050138 15879675

[B67] Cocks TM Fong B Chow JM Anderson GP Frauman AG Goldie RG Henry PJ Carr MJ Hamilton JR Moffatt JD A protective role for protease-activated receptors in the airways Nature 1999 398 156 160 10.1038/18223 10086357

[B68] Morello S Vellecco V Roviezzo F Maffia P Cuzzocrea S Cirino G Cicala C A protective role for proteinase activated receptor 2 in airways of lipopolysaccharide-treated rats Biochem Pharmacol 2005 71 223 230 10.1016/j.bcp.2005.10.016 16300746

[B69] Kawao N Nagataki M Nagasawa K Kubo S Cushing K Wada T Sekiguchi F Ichida S Hollenberg MD MacNaughton WK Signal transduction for proteinase-activated receptor-2-triggered prostaglandin E2 formation in human lung epithelial cells J Pharmacol Exp Ther 2005 315 576 589 10.1124/jpet.105.089490 16120814

[B70] Lan RS Knight DA Stewart GA Henry PJ Role of PGE(2) in protease-activated receptor-1, -2 and −4 mediated relaxation in the mouse isolated trachea Br J Pharmacol 2001 132 93 100 10.1038/sj.bjp.0703776 11156565 PMC1572534

[B71] Henry PJ D'Aprile A Self G Hong T Mann TS Inhibitors of prostaglandin transport and metabolism augment protease-activated receptor-2-mediated increases in prostaglandin E2 levels and smooth muscle relaxation in mouse isolated trachea J Pharmacol Exp Ther 2005 314 995 1001 10.1124/jpet.105.086124 15937152

[B72] De Campo BA Henry PJ Stimulation of protease-activated receptor-2 inhibits airway eosinophilia, hyperresponsiveness and bronchoconstriction in a murine model of allergic inflammation Br J Pharmacol 2005 144 1100 1108 10.1038/sj.bjp.0706150 15700024 PMC1576095

[B73] Vancheri C Mastruzzo C Sortino MA Crimi N The lung as a privileged site for the beneficial actions of PGE2 Trends Immunol 2004 25 40 46 10.1016/j.it.2003.11.001 14698283

[B74] Maher SA Belvisi MG Prostanoids and the cough reflex Lung 2010 188 Suppl 1 S9 S12 19830488 10.1007/s00408-009-9190-2

[B75] Kay LJ Yeo WW Peachell PT Prostaglandin E2 activates EP2 receptors to inhibit human lung mast cell degranulation Br J Pharmacol 2006 147 707 713 10.1038/sj.bjp.0706664 16432506 PMC1751511

[B76] Takayama K García-Cardena G Sukhova GK Comander J Gimbrone MA Libby P Prostaglandin E2 suppresses chemokine production in human macrophages through the EP4 receptor J Biol Chem 2002 277 44147 44154 10.1074/jbc.M204810200 12215436

[B77] Sastre B del Pozo V Role of PGE2 in asthma and nonasthmatic eosinophilic bronchitis Mediators Inflamm 2012 2012 645383 22529528 10.1155/2012/645383 PMC3316983

[B78] Henry PJ The protease-activated receptor2 (PAR2)-prostaglandin E2-prostanoid EP receptor axis: a potential bronchoprotective unit in the respiratory tract? Eur J Pharmacol 2006 533 156 170 10.1016/j.ejphar.2005.12.051 16483565

[B79] Nagataki M Moriyuki K Sekiguchi F Kawabata A Evidence that PAR2-triggered prostaglandin E2 (PGE2) formation involves the ERK-cytosolic phospholipase A2-COX-1-microsomal PGE synthase-1 cascade in human lung epithelial cells Cell Biochem Funct 2008 26 279 282 10.1002/cbf.1434 17708577

[B80] Moriyuki K Sekiguchi F Matsubara K Nishikawa H Kawabata A Proteinase-activated receptor-2-triggered prostaglandin E(2) release, but not cyclooxygenase-2 upregulation, requires activation of the phosphatidylinositol 3-kinase/Akt / nuclear factor-kappaB pathway in human alveolar epithelial cells J Pharmacol Sci 2009 111 269 275 10.1254/jphs.09155FP 19881225

[B81] Moriyuki K Sekiguchi F Matsubara K Nishikawa H Kawabata A Curcumin Inhibits the proteinase-activated receptor-2-triggered prostaglandin E2 production by suppressing cyclooxygenase-2 upregulation and Akt-dependent activation of nuclear factor-κB in human lung epithelial cells J Pharmacol Sci 2010 114 225 229 10.1254/jphs.10126SC 20838026

[B82] Komatsu H Enjouji S Ito A Ohama T Sato K Prostaglandin E(2) inhibits proteinase-activated receptor 2-signal transduction through regulation of receptor internalization J Vet Med Sci 2013 75 255 261 10.1292/jvms.12-0365 23064451

[B83] Asokananthan N Graham PT Fink J Knight DA Bakker AJ McWilliam AS Thompson PJ Stewart GA Activation of protease-activated receptor (PAR)-1, PAR-2, and PAR-4 stimulates IL-6, IL-8, and prostaglandin E2 release from human respiratory epithelial cells J Immunol 2002 168 3577 3585 11907122 10.4049/jimmunol.168.7.3577

[B84] Lo HM Chen CL Tsai YJ Wu PH Wu WB Thrombin induces cyclooxygenase-2 expression and prostaglandin E2 release via PAR1 activation and ERK1/2- and p38 MAPK-dependent pathway in murine macrophages J Cell Biochem 2009 108 1143 1152 10.1002/jcb.22341 19739103

[B85] Sokolova E Hartig R Reiser G Downregulation of protease-activated receptor-1 in human lung fibroblasts is specifically mediated by the prostaglandin E receptor EP2 through cAMP elevation and protein kinase A FEBS J 2008 275 3669 3679 10.1111/j.1742-4658.2008.06511.x 18537828

[B86] Kalmes A Daum G Clowes AW EGFR transactivation in the regulation of SMC function Ann N Y Acad Sci 2001 947 42 54 discussion 54–55 11795306 10.1111/j.1749-6632.2001.tb03929.x

[B87] Prenzel N Zwick E Daub H Leserer M Abraham R Wallasch C Ullrich A EGF receptor transactivation by G-protein-coupled receptors requires metalloproteinase cleavage of proHB-EGF Nature 1999 402 884 888 10622253 10.1038/47260

[B88] Wang Z Wang M Carr BI Integrin alpha5-induced EGFR activation by prothrombin triggers hepatocyte apoptosis via the JNK signaling pathway J Cell Physiol 2008 216 551 557 10.1002/jcp.21429 18330891

[B89] Sabri A Short J Guo J Steinberg SF Protease-activated receptor-1-mediated DNA synthesis in cardiac fibroblast is via epidermal growth factor receptor transactivation: distinct PAR-1 signaling pathways in cardiac fibroblasts and cardiomyocytes Circ Res 2002 91 532 539 10.1161/01.RES.0000035242.96310.45 12242272

[B90] Sabri A Guo J Elouardighi H Darrow AL Andrade-Gordon P Steinberg SF Mechanisms of protease-activated receptor-4 actions in cardiomyocytes. Role of Src tyrosine kinase J Biol Chem 2003 278 11714 11720 10.1074/jbc.M213091200 12522105

[B91] Abdallah RT Keum JS El-Shewy HM Lee MH Wang B Gooz M Luttrell DK Luttrell LM Jaffa AA Plasma kallikrein promotes epidermal growth factor receptor transactivation and signaling in vascular smooth muscle through direct activation of protease-activated receptors J Biol Chem 2010 285 35206 35215 10.1074/jbc.M110.171769 20826789 PMC2966134

[B92] Al-Ani B Hewett P Cudmore M Fujisawa T Saifeddine M Williams H Ramma W Sissaoui S Jayaraman P Ohba M Activation of proteinase-activated receptor 2 stimulates soluble vascular endothelial growth factor receptor 1 release via epidermal growth factor receptor transactivation in endothelial cells Hypertension 2010 55 689-U641 20124108 10.1161/HYPERTENSIONAHA.109.136333

[B93] Oikonomopoulou K Hansen KK Saifeddine M Tea I Blaber M Blaber SI Scarisbrick I Andrade-Gordon P Cottrell GS Bunnett NW Proteinase-activated receptors, targets for kallikrein signaling J Biol Chem 2006 281 32095 32112 10.1074/jbc.M513138200 16885167

[B94] Oikonomopoulou K Hansen K Saifeddine M Vergnolle N Tea I Diamandis E Hollenberg M Proteinase-mediated cell signalling: targeting proteinase-activated receptors (PARs) by kallikreins and more Biol Chem 2006 387 677 685 16800728 10.1515/BC.2006.086

[B95] Tokumaru S Higashiyama S Endo T Nakagawa T Miyagawa JI Yamamori K Hanakawa Y Ohmoto H Yoshino K Shirakata Y Ectodomain shedding of epidermal growth factor receptor ligands is required for keratinocyte migration in cutaneous wound healing J Cell Biol 2000 151 209 220 10.1083/jcb.151.2.209 11038170 PMC2192647

[B96] Gao L Chao L Chao J A novel signaling pathway of tissue kallikrein in promoting keratinocyte migration: activation of proteinase-activated receptor 1 and epidermal growth factor receptor Exp Cell Res 2010 316 376 389 10.1016/j.yexcr.2009.10.022 19879874 PMC2812679

[B97] Huang CY Chen SY Tsai HC Hsu HC Tang CH Thrombin induces epidermal growth factor receptor transactivation and CCL2 expression in human osteoblasts Arthritis Rheum 2012 64 3344 3354 10.1002/art.34557 22674286

[B98] Xia M Sui Z Recent developments in CCR2 antagonists Expert Opin Ther Pat 2009 19 295 303 10.1517/13543770902755129 19441905

[B99] Russell FA Zhan S Dumas A Lagarde S Pouliot M McDougall JJ The pronociceptive effect of proteinase-activated receptor-4 stimulation in rat knee joints is dependent on mast cell activation Pain 2011 152 354 360 10.1016/j.pain.2010.10.038 21238854

[B100] Russell FA McDougall JJ Proteinase activated receptor (PAR) involvement in mediating arthritis pain and inflammation Inflamm Res 2009 58 119 126 10.1007/s00011-009-8087-0 19184346

[B101] Russell FA Schuelert N Veldhoen VE Hollenberg MD McDougall JJ Proteinase-activated receptor-2 (PAR(2) ) activation sensitises primary afferents and causes leukocyte rolling and adherence in the rat knee joint Br J Pharmacol 2012 167 1665 1678 10.1111/j.1476-5381.2012.02120.x 22849826 PMC3525869

[B102] Russell FA Veldhoen VE Tchitchkan D McDougall JJ Proteinase-activated receptor-4 (PAR4) activation leads to sensitization of rat joint primary afferents via a bradykinin B2 receptor-dependent mechanism J Neurophysiol 2010 103 155 163 10.1152/jn.00486.2009 19889854

[B103] Kelso EB Lockhart JC Hembrough T Dunning L Plevin R Hollenberg MD Sommerhoff CP McLean JS Ferrell WR Therapeutic promise of proteinase-activated receptor-2 antagonism in joint inflammation J Pharmacol Exp Ther 2006 316 1017 1024 16260582 10.1124/jpet.105.093807

[B104] Howell DC Johns RH Lasky JA Shan B Scotton CJ Laurent GJ Chambers RC Absence of proteinase-activated receptor-1 signaling affords protection from bleomycin-induced lung inflammation and fibrosis Am J Pathol 2005 166 1353 1365 10.1016/S0002-9440(10)62354-1 15855637 PMC1606391

[B105] Yagi Y Otani H Ando S Oshiro A Kawai K Nishikawa H Araki H Fukuhara S Inagaki C Involvement of Rho signaling in PAR2-mediated regulation of neutrophil adhesion to lung epithelial cells Eur J Pharmacol 2006 536 19 27 10.1016/j.ejphar.2006.02.024 16564523

[B106] Arizmendi NG Abel M Mihara K Davidson C Polley D Nadeem A El Mays T Gilmore BF Walker B Gordon JR Mucosal allergic sensitization to cockroach allergens is dependent on proteinase activity and proteinase-activated receptor-2 activation J Immunol 2011 186 3164 3172 10.4049/jimmunol.0903812 21270400

[B107] Nichols HL Saffeddine M Theriot BS Hegde A Polley D El-Mays T Vliagoftis H Hollenberg MD Wilson EH Walker JK Defea KA β-Arrestin-2 mediates the proinflammatory effects of proteinase-activated receptor-2 in the airway Proc Natl Acad Sci U S A 2012 109 16660 16665 10.1073/pnas.1208881109 23012429 PMC3478622

[B108] Ando S Otani H Yagi Y Kawai K Araki H Fukuhara S Inagaki C Proteinase-activated receptor 4 stimulation-induced epithelial-mesenchymal transition in alveolar epithelial cells Respir Res 2007 8 31 10.1186/1465-9921-8-31 17433115 PMC1855055

[B109] Moriyuki K Nagataki M Sekiguchi F Nishikawa H Kawabata A Signal transduction for formation/release of interleukin-8 caused by a PAR2-activating peptide in human lung epithelial cells Regul Pept 2008 145 42 48 10.1016/j.regpep.2007.08.002 17854923

[B110] Bhola NE Grandis JR Crosstalk between G-protein-coupled receptors and epidermal growth factor receptor in cancer Front Biosci 2008 13 1857 1865 10.2741/2805 17981673

[B111] Bergmann S Junker K Henklein P Hollenberg MD Settmacher U Kaufmann R PAR-type thrombin receptors in renal carcinoma cells: PAR(1)-mediated EGFR activation promotes cell migration Oncol Rep 2006 15 889 893 16525676

[B112] Darmoul D Gratio V Devaud H Peiretti F Laburthe M Activation of proteinase-activated receptor 1 promotes human colon cancer cell proliferation through epidermal growth factor receptor transactivation Mol Cancer Res 2004 2 514 522 15383630

[B113] Darmoul D Gratio V Devaud H Laburthe M Protease-activated receptor 2 in colon cancer: trypsin-induced MAPK phosphorylation and cell proliferation are mediated by epidermal growth factor receptor transactivation J Biol Chem 2004 279 20927 20934 10.1074/jbc.M401430200 15010475

[B114] Jarry A Dorso L Gratio V Forgue-Lafitte M Laburthe M Laboisse C Darmoul D PAR-2 activation increases human intestinal mucin secretion through EGFR transactivation Biochem Biophys Res Commun 2007 364 689 694 10.1016/j.bbrc.2007.10.073 18028876

[B115] van der Merwe JQ Hollenberg MD MacNaughton WK EGF receptor transactivation and MAP kinase mediate proteinase-activated receptor-2-induced chloride secretion in intestinal epithelial cells Am J Physiol Gastrointest Liver Physiol 2008 294 G441 G451 18032480 10.1152/ajpgi.00303.2007

[B116] Caruso R Pallone F Fina D Gioia V Peluso I Caprioli F Stolfi C Perfetti A Spagnoli L Palmieri G Protease-activated receptor-2 activation in gastric cancer cells promotes epidermal growth factor receptor trans-activation and proliferation Am J Pathol 2006 169 268 278 10.2353/ajpath.2006.050841 16816379 PMC1698759

[B117] Fujimoto D Hirono Y Goi T Katayama K Matsukawa S Yamaguchi A The activation of proteinase-activated receptor-1 (PAR1) mediates gastric cancer cell proliferation and invasion BMC Cancer 2010 10 443 10.1186/1471-2407-10-443 20723226 PMC2933627

[B118] Gratio V Walker F Lehy T Laburthe M Darmoul D Aberrant expression of proteinase-activated receptor 4 promotes colon cancer cell proliferation through a persistent signaling that involves Src and ErbB-2 kinase Int J Cancer 2009 124 1517 1525 10.1002/ijc.24070 19058300

[B119] Gratio V Loriot C Virca GD Oikonomopoulou K Walker F Diamandis EP Hollenberg MD Darmoul D Kallikrein-related peptidase 14 acts on proteinase-activated receptor 2 to induce signaling pathway in colon cancer cells Am J Pathol 2011 179 2625 2636 10.1016/j.ajpath.2011.07.016 21907696 PMC3204030

[B120] Chung H Hamza M Oikonomopoulou K Gratio V Saifeddine M Virca GD Diamandis EP Hollenberg MD Darmoul D Kallikrein-related peptidase signaling in colon carcinoma cells: targeting proteinase-activated receptors Biol Chem 2012 393 413 420 22505523 10.1515/bc-2011-231

[B121] Darmoul D Gratio V Devaud H Lehy T Laburthe M Aberrant expression and activation of the thrombin receptor protease-activated receptor-1 induces cell proliferation and motility in human colon cancer cells Am J Pathol 2003 162 1503 1513 10.1016/S0002-9440(10)64283-6 12707033 PMC1851194

[B122] Arora P Cuevas BD Russo A Johnson GL Trejo J Persistent transactivation of EGFR and ErbB2/HER2 by protease-activated receptor-1 promotes breast carcinoma cell invasion Oncogene 2008 27 4434 4445 10.1038/onc.2008.84 18372913 PMC2874884

[B123] Aggarwal BB Shishodia S Sandur SK Pandey MK Sethi G Inflammation and cancer: how hot is the link? Biochem Pharmacol 2006 72 1605 1621 10.1016/j.bcp.2006.06.029 16889756

[B124] Sethi G Shanmugam MK Ramachandran L Kumar AP Tergaonkar V Multifaceted link between cancer and inflammation Biosci Rep 2012 32 1 15 10.1042/BSR20100136 21981137

[B125] Rothmeier AS Ruf W Protease-activated receptor 2 signaling in inflammation Semin Immunopathol 2012 34 133 149 10.1007/s00281-011-0289-1 21971685

[B126] Kawabata A Matsunami M Sekiguchi F Gastrointestinal roles for proteinase-activated receptors in health and disease Br J Pharmacol 2008 153 Suppl 1 S230 S240 17994114 10.1038/sj.bjp.0707491 PMC2268065

[B127] Hirota CL Moreau F Iablokov V Dicay M Renaux B Hollenberg MD MacNaughton WK Epidermal growth factor receptor transactivation is required for proteinase-activated receptor-2-induced COX-2 expression in intestinal epithelial cells Am J Physiol Gastrointest Liver Physiol 2012 303 G111 G119 10.1152/ajpgi.00358.2011 22517768

[B128] Milligan G G protein-coupled receptor hetero-dimerization: contribution to pharmacology and function Br J Pharmacol 2009 158 5 14 10.1111/j.1476-5381.2009.00169.x 19309353 PMC2795239

[B129] Bouvier M Oligomerization of G-protein-coupled transmitter receptors Nat Rev Neurosci 2001 2 274 286 10.1038/35067575 11283750

[B130] George SR O'Dowd BF Lee SP G-protein-coupled receptor oligomerization and its potential for drug discovery Nat Rev Drug Discov 2002 1 808 820 10.1038/nrd913 12360258

[B131] Breitwieser GE G protein-coupled receptor oligomerization: implications for G protein activation and cell signaling Circ Res 2004 94 17 27 10.1161/01.RES.0000110420.68526.19 14715532

[B132] Jordan BA Devi LA G-protein-coupled receptor heterodimerization modulates receptor function Nature 1999 399 697 700 10.1038/21441 10385123 PMC3125690

[B133] Ferré S Baler R Bouvier M Caron MG Devi LA Durroux T Fuxe K George SR Javitch JA Lohse MJ Building a new conceptual framework for receptor heteromers Nat Chem Biol 2009 5 131 134 10.1038/nchembio0309-131 19219011 PMC2681085

[B134] Ritter SL Hall RA Fine-tuning of GPCR activity by receptor-interacting proteins Nat Rev Mol Cell Biol 2009 10 819 830 19935667 10.1038/nrm2803 PMC2825052

[B135] O'Brien PJ Prevost N Molino M Hollinger MK Woolkalis MJ Woulfe DS Brass LF Thrombin responses in human endothelial cells. Contributions from receptors other than PAR1 include the transactivation of PAR2 by thrombin-cleaved PAR1 J Biol Chem 2000 275 13502 13509 10.1074/jbc.275.18.13502 10788464

[B136] Chen J Ishii M Wang L Ishii K Coughlin SR Thrombin receptor activation. Confirmation of the intramolecular tethered liganding hypothesis and discovery of an alternative intermolecular liganding mode J Biol Chem 1994 269 16041 16045 8206902

[B137] Shi X Gangadharan B Brass L Ruf W Mueller B Protease-activated receptors (PAR1 and PAR2) contribute to tumor cell motility and metastasis Mol Cancer Res 2004 2 395 402 15280447

[B138] Kaufmann R Patt S Zieger M Kraft R Tausch S Henklein P Nowak G The two-receptor system PAR-1/PAR-4 mediates alpha-thrombin-induced [Ca(2+)](i) mobilization in human astrocytoma cells J Cancer Res Clin Oncol 2000 126 91 94 10.1007/PL00008481 10664248 PMC12165160

[B139] Vesey DA Cheung CW Kruger WA Poronnik P Gobe G Johnson DW Thrombin stimulates proinflammatory and proliferative responses in primary cultures of human proximal tubule cells Kidney Int 2005 67 1315 1329 10.1111/j.1523-1755.2005.00209.x 15780084

[B140] Damiano BP Cheung WM Santulli RJ Fung-Leung WP Ngo K Ye RD Darrow AL Derian CK de Garavilla L Andrade-Gordon P Cardiovascular responses mediated by protease-activated receptor-2 (PAR-2) and thrombin receptor (PAR-1) are distinguished in mice deficient in PAR-2 or PAR-1 J Pharmacol Exp Ther 1999 288 671 678 9918574

[B141] McEachron TA Pawlinski R Richards KL Church FC Mackman N Protease-activated receptors mediate crosstalk between coagulation and fibrinolysis Blood 2010 116 5037 5044 10.1182/blood-2010-06-293126 20736455 PMC3012597

[B142] Cottrell GS Amadesi S Grady EF Bunnett NW Trypsin IV, a novel agonist of protease-activated receptors 2 and 4 J Biol Chem 2004 279 13532 13539 10.1074/jbc.M312090200 14726524

[B143] Leger AJ Jacques SL Badar J Kaneider NC Derian CK Andrade-Gordon P Covic L Kuliopulos A Blocking the protease-activated receptor 1–4 heterodimer in platelet-mediated thrombosis Circulation 2006 113 1244 1254 10.1161/CIRCULATIONAHA.105.587758 16505172

[B144] Milligan G G protein-coupled receptor dimerization: function and ligand pharmacology Mol Pharmacol 2004 66 1 7 10.1124/mol.104.000497. 15213289

[B145] James JR Oliveira MI Carmo AM Iaboni A Davis SJ A rigorous experimental framework for detecting protein oligomerization using bioluminescence resonance energy transfer Nat Methods 2006 3 1001 1006 10.1038/nmeth978 17086179

[B146] Overton MC Blumer KJ Use of fluorescence resonance energy transfer to analyze oligomerization of G-protein-coupled receptors expressed in yeast Methods 2002 27 324 332 10.1016/S1046-2023(02)00090-7 12217648

[B147] Cottet M Albizu L Comps-Agrar L Trinquet E Pin JP Mouillac B Durroux T Time resolved FRET strategy with fluorescent ligands to analyze receptor interactions in native tissues: application to GPCR oligomerization Methods Mol Biol 2011 746 373 387 10.1007/978-1-61779-126-0_21 21607869

[B148] Comps-Agrar L Maurel D Rondard P Pin JP Trinquet E Prézeau L Cell-surface protein-protein interaction analysis with time-resolved FRET and snap-tag technologies: application to G protein-coupled receptor oligomerization Methods Mol Biol 2011 756 201 214 10.1007/978-1-61779-160-4_10 21870227

[B149] Kaneider NC Leger AJ Agarwal A Nguyen N Perides G Derian C Covic L Kuliopulos A 'Role reversal' for the receptor PAR1 in sepsis-induced vascular damage Nat Immunol 2007 8 1303 1312 10.1038/ni1525 17965715 PMC3059149

[B150] Sevigny LM Austin KM Zhang P Kasuda S Koukos G Sharifi S Covic L Kuliopulos A Protease-activated receptor-2 modulates protease-activated receptor-1-driven neointimal hyperplasia Arterioscler Thromb Vasc Biol 2011 31 e100 e106 10.1161/ATVBAHA.111.238261 21940952 PMC3241440

[B151] Soh UJ Trejo J Activated protein C promotes protease-activated receptor-1 cytoprotective signaling through β-arrestin and dishevelled-2 scaffolds Proc Natl Acad Sci U S A 2011 108 E1372 E1380 10.1073/pnas.1112482108 22106258 PMC3250136

[B152] Lin H Trejo J Transactivation of the PAR1-PAR2 heterodimer by thrombin elicits β-arrestin-mediated endosomal signaling J Biol Chem 2013 288 11203 11215 10.1074/jbc.M112.439950 23476015 PMC3630854

[B153] Kahn ML Zheng YW Huang W Bigornia V Zeng D Moff S Farese RV Tam C Coughlin SR A dual thrombin receptor system for platelet activation Nature 1998 394 690 694 10.1038/29325 9716134

[B154] Kahn ML Nakanishi-Matsui M Shapiro MJ Ishihara H Coughlin SR Protease-activated receptors 1 and 4 mediate activation of human platelets by thrombin J Clin Invest 1999 103 879 887 10.1172/JCI6042 10079109 PMC408153

[B155] Nieman MT Protease-activated receptor 4 uses anionic residues to interact with alpha-thrombin in the absence or presence of protease-activated receptor 1 Biochemistry 2008 47 13279 13286 10.1021/bi801334s 19053259

[B156] Covic L Gresser AL Kuliopulos A Biphasic kinetics of activation and signaling for PAR1 and PAR4 thrombin receptors in platelets Biochemistry 2000 39 5458 5467 10.1021/bi9927078 10820018

[B157] Shapiro MJ Weiss EJ Faruqi TR Coughlin SR Protease-activated receptors 1 and 4 are shut off with distinct kinetics after activation by thrombin J Biol Chem 2000 275 25216 25221 10.1074/jbc.M004589200 10837487

[B158] Kaufmann R Rahn S Pollrich K Hertel J Dittmar Y Hommann M Henklein P Biskup C Westermann M Hollenberg M Settmacher U Thrombin-mediated hepatocellular carcinoma cell migration: cooperative action via proteinase-activated receptors 1 and 4 J Cell Physiol 2007 211 699 707 10.1002/jcp.21027 17323377

[B159] Bah A Chen Z Bush-Pelc LA Mathews FS Di Cera E Crystal structures of murine thrombin in complex with the extracellular fragments of murine protease-activated receptors PAR3 and PAR4 Proc Natl Acad Sci USA 2007 104 11603 11608 10.1073/pnas.0704409104 17606903 PMC1913866

[B160] Nelken NA Soifer SJ O'Keefe J Vu TK Charo IF Coughlin SR Thrombin receptor expression in normal and atherosclerotic human arteries J Clin Invest 1992 90 1614 1621 10.1172/JCI116031 1328304 PMC443210

[B161] Mirza H Yatsula V Bahou WF The proteinase activated receptor-2 (PAR-2) mediates mitogenic responses in human vascular endothelial cells J Clin Invest 1996 97 1705 1714 10.1172/JCI118597 8601636 PMC507235

[B162] Schmidt VA Nierman WC Maglott DR Cupit LD Moskowitz KA Wainer JA Bahou WF The human proteinase-activated receptor-3 (PAR-3) gene. Identification within a Par gene cluster and characterization in vascular endothelial cells and platelets J Biol Chem 1998 273 15061 15068 10.1074/jbc.273.24.15061 9614115

[B163] Kataoka H Hamilton JR McKemy DD Camerer E Zheng YW Cheng A Griffin C Coughlin SR Protease-activated receptors 1 and 4 mediate thrombin signaling in endothelial cells Blood 2003 102 3224 3231 10.1182/blood-2003-04-1130 12869501

[B164] Fujiwara M Jin E Ghazizadeh M Kawanami O Activation of PAR4 induces a distinct actin fiber formation via p38 MAPK in human lung endothelial cells J Histochem Cytochem 2005 53 1121 1129 10.1369/jhc.4A6592.2005 15923365

[B165] McLaughlin JN Patterson MM Malik AB Protease-activated receptor-3 (PAR3) regulates PAR1 signaling by receptor dimerization Proc Natl Acad Sci USA 2007 104 5662 5667 10.1073/pnas.0700763104 17376866 PMC1838494

[B166] Cunningham MR McIntosh KA Pediani JD Robben J Cooke AE Nilsson M Gould GW Mundell S Milligan G Plevin R Novel role for proteinase-activated receptor 2 (PAR2) in membrane trafficking of proteinase-activated receptor 4 (PAR4) J Biol Chem 2012 287 16656 16669 10.1074/jbc.M111.315911 22411985 PMC3351358

[B167] de la Fuente M Noble DN Verma S Nieman MT Mapping human protease-activated receptor 4 (PAR4) homodimer interface to transmembrane helix 4 J Biol Chem 2012 287 10414 10423 10.1074/jbc.M112.341438 22318735 PMC3322995

[B168] Dorsam RT Tuluc M Kunapuli SP Role of protease-activated and ADP receptor subtypes in thrombin generation on human platelets J Thromb Haemost 2004 2 804 812 10.1111/j.1538-7836.2004.00692.x 15099288

[B169] Shankar H Garcia A Prabhakar J Kim S Kunapuli SP P2Y12 receptor-mediated potentiation of thrombin-induced thromboxane A2 generation in platelets occurs through regulation of Erk1/2 activation J Thromb Haemost 2006 4 638 647 10.1111/j.1538-7836.2006.01789.x 16460446

[B170] Henriksen RA Hanks VK PAR-4 agonist AYPGKF stimulates thromboxane production by human platelets Arterioscler Thromb Vasc Biol 2002 22 861 866 10.1161/01.ATV.0000014742.56572.25 12006403

[B171] Henriksen RA Samokhin GP Tracy PB Thrombin-induced thromboxane synthesis by human platelets. Properties of anion binding exosite I-independent receptor Arterioscler Thromb Vasc Biol 1997 17 3519 3526 10.1161/01.ATV.17.12.3519 9437201

[B172] Wu CC Hwang TL Liao CH Kuo SC Lee FY Teng CM The role of PAR4 in thrombin-induced thromboxane production in human platelets Thromb Haemost 2003 90 299 308 12888878 10.1160/TH03-02-0103

[B173] Li D D'Angelo L Chavez M Woulfe DS Arrestin-2 differentially regulates PAR4 and ADP receptor signaling in platelets J Biol Chem 2011 286 3805 3814 10.1074/jbc.M110.118018 21106537 PMC3030382

[B174] Hollenberg MD Saifeddine M Sandhu S Houle S Vergnolle N Proteinase-activated receptor-4: evaluation of tethered ligand-derived peptides as probes for receptor function and as inflammatory agonists in vivo Br J Pharmacol 2004 143 443 454 10.1038/sj.bjp.0705946 15451771 PMC1575414

[B175] Vergnolle N Derian CK D'Andrea MR Steinhoff M Andrade-Gordon P Characterization of thrombin-induced leukocyte rolling and adherence: a potential proinflammatory role for proteinase-activated receptor-4 J Immunol 2002 169 1467 1473 12133973 10.4049/jimmunol.169.3.1467

[B176] Houle S Papez MD Ferazzini M Hollenberg MD Vergnolle N Neutrophils and the kallikrein-kinin system in proteinase-activated receptor 4-mediated inflammation in rodents Br J Pharmacol 2005 146 670 678 10.1038/sj.bjp.0706371 16100525 PMC1751199

[B177] McDougall JJ Zhang C Cellars L Joubert E Dixon CM Vergnolle N Triggering of proteinase-activated receptor 4 leads to joint pain and inflammation in mice Arthritis Rheum 2009 60 728 737 10.1002/art.24300 19248120

[B178] Hatton MW Moar SL Richardson M Deendothelialization in vivo initiates a thrombogenic reaction at the rabbit aorta surface. Correlation of uptake of fibrinogen and antithrombin III with thrombin generation by the exposed subendothelium Am J Pathol 1989 135 499 508 2782381 PMC1879878

[B179] Herbert JM Lamarche I Dol F Induction of vascular smooth muscle cell growth by selective activation of the thrombin receptor. Effect of heparin FEBS Lett 1992 301 155 158 10.1016/0014-5793(92)81237-G 1314739

[B180] McNamara CA Sarembock IJ Gimple LW Fenton JW Coughlin SR Owens GK Thrombin stimulates proliferation of cultured rat aortic smooth muscle cells by a proteolytically activated receptor J Clin Invest 1993 91 94 98 10.1172/JCI116206 8380817 PMC330000

[B181] Hirano K The roles of proteinase-activated receptors in the vascular physiology and pathophysiology Arterioscler Thromb Vasc Biol 2007 27 27 36 10.1161/01.ATV.0000251995.73307.2d 17095716

[B182] Fukunaga R Hirano K Hirano M Niiro N Nishimura J Maehara Y Kanaide H Upregulation of proteinase-activated receptors and hypercontractile responses precede development of arterial lesions after balloon injury Am J Physiol Heart Circ Physiol 2006 291 H2388 H2395 10.1152/ajpheart.01313.2005 16844909

[B183] Wilcox JN Rodriguez J Subramanian R Ollerenshaw J Zhong C Hayzer DJ Horaist C Hanson SR Lumsden A Salam TA Characterization of thrombin receptor expression during vascular lesion formation Circ Res 1994 75 1029 1038 10.1161/01.RES.75.6.1029 7955141

[B184] Schini-Kerth VB Fisslthaler B Van Obberghen-Schilling E Busse R Serotonin stimulates the expression of thrombin receptors in cultured vascular smooth muscle cells. Role of protein kinase C and protein tyrosine kinases Circulation 1996 93 2170 2177 10.1161/01.CIR.93.12.2170 8925586

[B185] Fisslthaler B Schini-Kerth VB Fleming I Busse R Thrombin receptor expression is increased by angiotensin II in cultured and native vascular smooth muscle cells Cardiovasc Res 1998 38 263 271 10.1016/S0008-6363(97)00296-4 9683930

[B186] Capers Q Laursen JB Fukui T Rajagopalan S Mori I Lou P Freeman BA Berrington WR Griendling KK Harrison DG Vascular thrombin receptor regulation in hypertensive rats Circ Res 1997 80 838 844 10.1161/01.RES.80.6.838 9168786

[B187] Rallabhandi P Nhu QM Toshchakov VY Piao W Medvedev AE Hollenberg MD Fasano A Vogel SN Analysis of proteinase-activated receptor 2 and TLR4 signal transduction: a novel paradigm for receptor cooperativity J Biol Chem 2008 283 24314 24325 10.1074/jbc.M804800200 18622013 PMC2528983

[B188] Nhu QM Shirey K Teijaro JR Farber DL Netzel-Arnett S Antalis TM Fasano A Vogel SN Novel signaling interactions between proteinase-activated receptor 2 and Toll-like receptors in vitro and in vivo Mucosal Immunol 2010 3 29 39 10.1038/mi.2009.120 19865078 PMC2851245

[B189] Nhu QM Shirey KA Pennini ME Stiltz J Vogel SN Proteinase-activated receptor 2 activation promotes an anti-inflammatory and alternatively activated phenotype in LPS-stimulated murine macrophages Innate Immun 2012 18 193 203 10.1177/1753425910395044 21239455 PMC3724520

[B190] Zhou B Zhou H Ling S Guo D Yan Y Zhou F Wu Y Activation of PAR2 or/and TLR4 promotes SW620 cell proliferation and migration via phosphorylation of ERK1/2 Oncol Rep 2011 25 503 511 21152870 10.3892/or.2010.1077

[B191] Moraes TJ Martin R Plumb JD Vachon E Cameron CM Danesh A Kelvin DJ Ruf W Downey GP Role of PAR2 in murine pulmonary pseudomonal infection Am J Physiol Lung Cell Mol Physiol 2008 294 L368 L377 18083764 10.1152/ajplung.00036.2007

[B192] Moretti S Bellocchio S Bonifazi P Bozza S Zelante T Bistoni F Romani L The contribution of PARs to inflammation and immunity to fungi Mucosal Immunol 2008 1 156 168 10.1038/mi.2007.13 19079173

[B193] Bucci M Vellecco V Harrington L Brancaleone V Roviezzo F Mattace Raso G Ianaro A Lungarella G De Palma R Meli R Cirino G Cross-talk between toll-like receptor 4 (TLR4) and proteinase-activated receptor 2 (PAR(2)) is involved in vascular function Br J Pharmacol 2013 168 411 420 10.1111/j.1476-5381.2012.02205.x 22957757 PMC3572567

[B194] Kersse K Bertrand MJ Lamkanfi M Vandenabeele P NOD-like receptors and the innate immune system: coping with danger, damage and death Cytokine Growth Factor Rev 2011 22 257 276 10.1016/j.cytogfr.2011.09.003 21996492

[B195] Uehara A Imamura T Potempa J Travis J Takada H Gingipains from Porphyromonas gingivalis synergistically induce the production of proinflammatory cytokines through protease-activated receptors with Toll-like receptor and NOD1/2 ligands in human monocytic cells Cell Microbiol 2008 10 1181 1189 10.1111/j.1462-5822.2008.01119.x 18182086

[B196] Gingrich MB Junge CE Lyuboslavsky P Traynelis SF Potentiation of NMDA receptor function by the serine protease thrombin J Neurosci 2000 20 4582 4595 10844028 10.1523/JNEUROSCI.20-12-04582.2000 PMC6772448

[B197] Vivien D Buisson A Serine protease inhibitors: novel therapeutic targets for stroke? J Cereb Blood Flow Metab 2000 20 755 764 10826525 10.1097/00004647-200005000-00001

[B198] Matsuoka H Hamada R Role of thrombin in CNS damage associated with intracerebral haemorrhage: opportunity for pharmacological intervention? CNS Drugs 2002 16 509 516 10.2165/00023210-200216080-00001 12096932

[B199] Xi G Reiser G Keep RF The role of thrombin and thrombin receptors in ischemic, hemorrhagic and traumatic brain injury: deleterious or protective? J Neurochem 2003 84 3 9 12485396 10.1046/j.1471-4159.2003.01268.x

[B200] Ruf W PAR1 signaling: more good than harm? Nat Med 2003 9 258 260 10.1038/nm0303-258 12612568

[B201] Sheehan JJ Tsirka SE Fibrin-modifying serine proteases thrombin, tPA, and plasmin in ischemic stroke: a review Glia 2005 50 340 350 10.1002/glia.20150 15846799

[B202] Lee CJ Mannaioni G Yuan H Woo DH Gingrich MB Traynelis SF Astrocytic control of synaptic NMDA receptors J Physiol 2007 581 1057 1081 10.1113/jphysiol.2007.130377 17412766 PMC2170820

[B203] Hollmann M O'Shea-Greenfield A Rogers SW Heinemann S Cloning by functional expression of a member of the glutamate receptor family Nature 1989 342 643 648 10.1038/342643a0 2480522

[B204] Mannaioni G Orr AG Hamill CE Yuan H Pedone KH McCoy KL Berlinguer Palmini R Junge CE Lee CJ Yepes M Plasmin potentiates synaptic N-methyl-D-aspartate receptor function in hippocampal neurons through activation of protease-activated receptor-1 J Biol Chem 2008 283 20600 20611 10.1074/jbc.M803015200 18474593 PMC2459301

[B205] Nagai T Ito M Nakamichi N Mizoguchi H Kamei H Fukakusa A Nabeshima T Takuma K Yamada K The rewards of nicotine: regulation by tissue plasminogen activator-plasmin system through protease activated receptor-1 J Neurosci 2006 26 12374 12383 10.1523/JNEUROSCI.3139-06.2006 17122062 PMC6675418

[B206] Hamill CE Goldshmidt A Nicole O McKeon RJ Brat DJ Traynelis SF Special lecture: glial reactivity after damage: implications for scar formation and neuronal recovery Clin Neurosurg 2005 52 29 44 16626052

[B207] Hamill CE Mannaioni G Lyuboslavsky P Sastre AA Traynelis SF Protease-activated receptor 1-dependent neuronal damage involves NMDA receptor function Exp Neurol 2009 217 136 146 10.1016/j.expneurol.2009.01.023 19416668 PMC2679858

[B208] Han KS Mannaioni G Hamill CE Lee J Junge CE Lee CJ Traynelis SF Activation of protease activated receptor 1 increases the excitability of the dentate granule neurons of hippocampus Mol Brain 2011 4 32 10.1186/1756-6606-4-32 21827709 PMC3170262

[B209] Fields RD Burnstock G Purinergic signalling in neuron-glia interactions Nat Rev Neurosci 2006 7 423 436 10.1038/nrn1928 16715052 PMC2062484

[B210] Shigetomi E Bowser DN Sofroniew MV Khakh BS Two forms of astrocyte calcium excitability have distinct effects on NMDA receptor-mediated slow inward currents in pyramidal neurons J Neurosci 2008 28 6659 6663 10.1523/JNEUROSCI.1717-08.2008 18579739 PMC2866443

[B211] Boven LA Vergnolle N Henry SD Silva C Imai Y Holden J Warren K Hollenberg MD Power C Up-regulation of proteinase-activated receptor 1 expression in astrocytes during HIV encephalitis J Immunol 2003 170 2638 2646 12594292 10.4049/jimmunol.170.5.2638

[B212] Gan J Greenwood SM Cobb SR Bushell TJ Indirect modulation of neuronal excitability and synaptic transmission in the hippocampus by activation of proteinase-activated receptor-2 Br J Pharmacol 2011 163 984 994 10.1111/j.1476-5381.2011.01293.x 21366553 PMC3130945

[B213] Grenegård M Vretenbrant-Oberg K Nylander M Désilets S Lindström EG Larsson A Ramström I Ramström S Lindahl TL The ATP-gated P2X1 receptor plays a pivotal role in activation of aspirin-treated platelets by thrombin and epinephrine J Biol Chem 2008 283 18493 18504 10.1074/jbc.M800358200 18480058

[B214] Ramsey IS Delling M Clapham DE An introduction to TRP channels Annu Rev Physiol 2006 68 619 647 10.1146/annurev.physiol.68.040204.100431 16460286

[B215] Wu LJ Sweet TB Clapham DE International union of basic and clinical pharmacology. LXXVI. Current progress in the mammalian TRP ion channel family Pharmacol Rev 2010 62 381 404 10.1124/pr.110.002725 20716668 PMC2964900

[B216] Steinhoff M Vergnolle N Young SH Tognetto M Amadesi S Ennes HS Trevisani M Hollenberg MD Wallace JL Caughey GH Agonists of proteinase-activated receptor 2 induce inflammation by a neurogenic mechanism Nat Med 2000 6 151 158 10.1038/72247 10655102

[B217] de Garavilla L Vergnolle N Young SH Ennes H Steinhoff M Ossovskaya VS D'Andrea MR Mayer EA Wallace JL Hollenberg MD Agonists of proteinase-activated receptor 1 induce plasma extravasation by a neurogenic mechanism Br J Pharmacol 2001 133 975 987 10.1038/sj.bjp.0704152 11487506 PMC1572861

[B218] Hoogerwerf WA Zou L Shenoy M Sun D Micci MA Lee-Hellmich H Xiao SY Winston JH Pasricha PJ The proteinase-activated receptor 2 is involved in nociception J Neurosci 2001 21 9036 9042 11698614 10.1523/JNEUROSCI.21-22-09036.2001 PMC6762290

[B219] Dai Y Moriyama T Higashi T Togashi K Kobayashi K Yamanaka H Tominaga M Noguchi K Proteinase-activated receptor 2-mediated potentiation of transient receptor potential vanilloid subfamily 1 activity reveals a mechanism for proteinase-induced inflammatory pain J Neurosci 2004 24 4293 4299 10.1523/JNEUROSCI.0454-04.2004 15128843 PMC6729433

[B220] Amadesi S Nie J Vergnolle N Cottrell GS Grady EF Trevisani M Manni C Geppetti P McRoberts JA Ennes H Protease-activated receptor 2 sensitizes the capsaicin receptor transient receptor potential vanilloid receptor 1 to induce hyperalgesia J Neurosci 2004 24 4300 4312 10.1523/JNEUROSCI.5679-03.2004 15128844 PMC6729438

[B221] Kawabata A Kawao N Kuroda R Tanaka A Itoh H Nishikawa H Peripheral PAR-2 triggers thermal hyperalgesia and nociceptive responses in rats Neuroreport 2001 12 715 719 10.1097/00001756-200103260-00020 11277570

[B222] Grant A Amadesi S Bunnett NW WB L, Heller S Protease-activated receptors: Mechanism by which proteases sensitize TRPV channels to induce neurogenic inflammation and pain TRP ion channel function in sensory transduction and cellular signaling cascades 2007 Boca Raton (FL): CRC Press 21204493

[B223] Cervero F Laird JM Understanding the signaling and transmission of visceral nociceptive events J Neurobiol 2004 61 45 54 10.1002/neu.20084 15362152

[B224] Stucky CL Dubin AE Jeske NA Malin SA McKemy DD Story GM Roles of transient receptor potential channels in pain Brain Res Rev 2009 60 2 23 10.1016/j.brainresrev.2008.12.018 19203589 PMC2683630

[B225] Amadesi S Cottrell GS Divino L Chapman K Grady EF Bautista F Karanjia R Barajas-Lopez C Vanner S Vergnolle N Bunnett NW Protease-activated receptor 2 sensitizes TRPV1 by protein kinase Cepsilon- and A-dependent mechanisms in rats and mice J Physiol 2006 575 555 571 10.1113/jphysiol.2006.111534 16793902 PMC1819458

[B226] Nishimura S Ishikura H Matsunami M Shinozaki Y Sekiguchi F Naruse M Kitamura T Akashi R Matsumura K Kawabata A The proteinase/proteinase-activated receptor-2/transient receptor potential vanilloid-1 cascade impacts pancreatic pain in mice Life Sci 2010 87 643 650 10.1016/j.lfs.2010.09.030 20932849

[B227] Zhang W Gao J Zhao T Wei L Wu W Bai Y Zou D Li Z Proteinase-activated receptor 2 mediates thermal hyperalgesia and is upregulated in a rat model of chronic pancreatitis Pancreas 2011 40 300 307 10.1097/MPA.0b013e318201cbc1 21311307

[B228] Suckow SK Anderson EM Caudle RM Lesioning of TRPV1 expressing primary afferent neurons prevents PAR-2 induced motility, but not mechanical hypersensitivity in the rat colon Neurogastroenterol Motil 2012 24 e125 e135 10.1111/j.1365-2982.2011.01848.x 22168801 PMC3276722

[B229] Jiang R Zatta A Kin H Wang N Reeves JG Mykytenko J Deneve J Zhao ZQ Guyton RA Vinten-Johansen J PAR-2 activation at the time of reperfusion salvages myocardium via an ERK1/2 pathway in in vivo rat hearts Am J Physiol Heart Circ Physiol 2007 293 H2845 H2852 10.1152/ajpheart.00209.2007 17720772

[B230] Napoli C Cicala C Wallace JL de Nigris F Santagada V Caliendo G Franconi F Ignarro LJ Cirino G Protease-activated receptor-2 modulates myocardial ischemia-reperfusion injury in the rat heart Proc Natl Acad Sci USA 2000 97 3678 3683 10.1073/pnas.97.7.3678 10737808 PMC16299

[B231] Napoli C De Nigris F Cicala C Wallace JL Caliendo G Condorelli M Santagada V Cirino G Protease-activated receptor-2 activation improves efficiency of experimental ischemic preconditioning Am J Physiol Heart Circ Physiol 2002 282 H2004 H2010 12003804 10.1152/ajpheart.00909.2001

[B232] McLean PG Aston D Sarkar D Ahluwalia A Protease-activated receptor-2 activation causes EDHF-like coronary vasodilation: selective preservation in ischemia/reperfusion injury: involvement of lipoxygenase products, VR1 receptors, and C-fibers Circ Res 2002 90 465 472 10.1161/hh0402.105372 11884377

[B233] Zhong B Wang DH Protease-activated receptor 2-mediated protection of myocardial ischemia-reperfusion injury: role of transient receptor potential vanilloid receptors Am J Physiol Regul Integr Comp Physiol 2009 297 R1681 R1690 10.1152/ajpregu.90746.2008 19812353 PMC2803628

[B234] Vellani V Kinsey AM Prandini M Hechtfischer SC Reeh P Magherini PC Giacomoni C McNaughton PA Protease activated receptors 1 and 4 sensitize TRPV1 in nociceptive neurones Mol Pain 2010 6 61 10.1186/1744-8069-6-61 20875131 PMC2956715

[B235] Poole DP Amadesi S Veldhuis NA Abogadie FC Lieu T Darby W Liedtke W Lew MJ McIntyre P Bunnett NW Protease-activated receptor 2 (PAR2) protein and transient receptor potential vanilloid 4 (TRPV4) protein coupling is required for sustained inflammatory signaling J Biol Chem 2013 288 5790 5802 10.1074/jbc.M112.438184 23288842 PMC3581372

[B236] Chen Y Yang C Wang ZJ Proteinase-activated receptor 2 sensitizes transient receptor potential vanilloid 1, transient receptor potential vanilloid 4, and transient receptor potential ankyrin 1 in paclitaxel-induced neuropathic pain Neuroscience 2011 193 440 451 21763756 10.1016/j.neuroscience.2011.06.085

[B237] Soh UJ Dores MR Chen B Trejo J Signal transduction by protease-activated receptors Br J Pharmacol 2010 160 191 203 10.1111/j.1476-5381.2010.00705.x 20423334 PMC2874842

[B238] Dominguez M Dejgaard K Füllekrug J Dahan S Fazel A Paccaud JP Thomas DY Bergeron JJ Nilsson T gp25L/emp24/p24 protein family members of the cis-Golgi network bind both COP I and II coatomer J Cell Biol 1998 140 751 765 10.1083/jcb.140.4.751 9472029 PMC2141742

[B239] Carney GE Bowen NJ p24 Proteins, intracellular trafficking, and behavior: drosophila melanogaster provides insights and opportunities Biol Cell 2004 96 271 278 15145531 10.1016/j.biolcel.2004.01.004

[B240] Luo W Wang Y Reiser G p24A, a type I transmembrane protein, controls ARF1-dependent resensitization of protease-activated receptor-2 by influence on receptor trafficking J Biol Chem 2007 282 30246 30255 10.1074/jbc.M703205200 17693410

[B241] Luo W Wang Y Reiser G Proteinase-activated receptors, nucleotide P2Y receptors, and μ-opioid receptor-1B are under the control of the type I transmembrane proteins p23 and p24A in post-Golgi trafficking J Neurochem 2011 117 71 81 10.1111/j.1471-4159.2011.07173.x 21219331

[B242] Pin JP Neubig R Bouvier M Devi L Filizola M Javitch JA Lohse MJ Milligan G Palczewski K Parmentier M Spedding M International union of basic and clinical pharmacology. LXVII. Recommendations for the recognition and nomenclature of G protein-coupled receptor heteromultimers Pharmacol Rev 2007 59 5 13 10.1124/pr.59.1.5 17329545

[B243] Ayoub MA Pfleger KD Recent advances in bioluminescence resonance energy transfer technologies to study GPCR heteromerization Curr Opin Pharmacol 2010 10 44 52 10.1016/j.coph.2009.09.012 19897419

[B244] Milligan G The role of dimerisation in the cellular trafficking of G-protein-coupled receptors Curr Opin Pharmacol 2010 10 23 29 10.1016/j.coph.2009.09.010 19850521

[B245] Lohse MJ Dimerization in GPCR mobility and signaling Curr Opin Pharmacol 2010 10 53 58 10.1016/j.coph.2009.10.007 19910252

[B246] Rozenfeld R Devi LA Receptor heteromerization and drug discovery Trends Pharmacol Sci 2010 31 124 130 10.1016/j.tips.2009.11.008 20060175 PMC2834828

[B247] Kenakin T New concepts in pharmacological efficacy at 7TM receptors: IUPHAR review 2 Br J Pharmacol 2013 168 554 575 10.1111/j.1476-5381.2012.02223.x 22994528 PMC3579279

[B248] Barki-Harrington L Luttrell LM Rockman HA Dual inhibition of beta-adrenergic and angiotensin II receptors by a single antagonist: a functional role for receptor-receptor interaction in vivo Circulation 2003 108 1611 1618 10.1161/01.CIR.0000092166.30360.78 12963634

[B249] Zhang C Srinivasan Y Arlow DH Fung JJ Palmer D Zheng Y Green HF Pandey A Dror RO Shaw DE High-resolution crystal structure of human protease-activated receptor 1 Nature 2012 492 387 392 10.1038/nature11701 23222541 PMC3531875

[B250] Sevigny LM Zhang P Bohm A Lazarides K Perides G Covic L Kuliopulos A Interdicting protease-activated receptor-2-driven inflammation with cell-penetrating pepducins Proc Natl Acad Sci USA 2011 108 8491 8496 10.1073/pnas.1017091108 21536878 PMC3100971

[B251] Suen JY Barry GD Lohman RJ Halili MA Cotterell AJ Le GT Fairlie DP Modulating human proteinase activated receptor 2 with a novel antagonist (GB88) and agonist (GB110) Br J Pharmacol 2012 165 1413 1423 10.1111/j.1476-5381.2011.01610.x 21806599 PMC3372726

[B252] Lohman RJ Cotterell AJ Suen J Liu L Do AT Vesey DA Fairlie DP Antagonism of protease-activated receptor 2 protects against experimental colitis J Pharmacol Exp Ther 2012 340 256 265 10.1124/jpet.111.187062 22028393

[B253] Lohman RJ Cotterell AJ Barry GD Liu L Suen JY Vesey DA Fairlie DP An antagonist of human protease activated receptor-2 attenuates PAR2 signaling, macrophage activation, mast cell degranulation, and collagen-induced arthritis in rats FASEB J 2012 26 2877 2887 10.1096/fj.11-201004 22467762

[B254] Hollenberg M Mihara K Polley D Fairlie D Ramachandran R Biased signalling and proteinase-activated receptors (pars): targeting inflammatory disease Brit J Pharmacol 2013 in press 10.1111/bph.12544 PMC3952797 24354792

[B255] Han A Honours Dissertation 2008 Queensland: University of Queensland

[B256] Nagaraj NS Datta PK Targeting the transforming growth factor-beta signaling pathway in human cancer Expert Opin Investig Drugs 2010 19 77 91 10.1517/13543780903382609 20001556 PMC2796203

[B257] Kamato D Burch ML Osman N Zheng W Little PJ Therapeutic implications of endothelin and thrombin G-protein-coupled receptor transactivation of tyrosine and serine/threonine kinase cell surface receptors J Pharm Pharmacol 2013 65 465 473 10.1111/j.2042-7158.2012.01577.x 23488775

